# System analysis based on the pyroptosis-related genes identifies GSDMC as a novel therapy target for pancreatic adenocarcinoma

**DOI:** 10.1186/s12967-022-03632-z

**Published:** 2022-10-05

**Authors:** Cheng Yan, Yandie Niu, Feng Li, Wei Zhao, Liukai Ma

**Affiliations:** School of Pharmacy, Key Laboratory of Nano-Carbon Modified Film Technology of Henan Province, Diagnostic Laboratory of Animal Diseases, Xinxiang University, Xinxiang, 453000 Henan China

**Keywords:** Pancreatic adenocarcinoma, Pyroptosis, Prognostic model, Drug, GSDMC, Immune infiltration

## Abstract

**Background:**

Pancreatic adenocarcinoma (PAAD) is one of the most common malignant tumors of the digestive tract. Pyroptosis is a newly discovered programmed cell death that highly correlated with the prognosis of tumors. However, the prognostic value of pyroptosis in PAAD remains unclear.

**Methods:**

A total of 178 pancreatic cancer PAAD samples and 167 normal samples were obtained from The Cancer Genome Atlas (TCGA) and Genotype-Tissue Expression (GTEx) databases. The “DESeq2” R package was used to identify differntially expressed pyroptosis-related genes between normal pancreatic samples and PAAD samples. The prognostic model was established in TCGA cohort based on univariate Cox and the least absolute shrinkage and selection operator (LASSO) Cox regression analyses, which was validated in test set from Gene Expression Omnibus (GEO) cohort. Univariate independent prognostic analysis and multivariate independent prognostic analysis were used to determine whether the risk score can be used as an independent prognostic factor to predict the clinicopathological features of PAAD patients. A nomogram was used to predict the survival probability of PAAD patients, which could help in clinical decision-making. The R package "pRRophetic" was applied to calculate the drug sensitivity of each samples from high- and low-risk group. Tumor immune infiltration was investigated using an ESTIMATE algorithm. Finally, the pro‐tumor phenotype of GSDMC was explored in PANC-1 and CFPAC-1 cells.

**Result:**

On the basis of univariate Cox and LASSO regression analyses, we constructed a risk model with identified five pyroptosis-related genes (IL18, CASP4, NLRP1, GSDMC, and NLRP2), which was validated in the test set. The PAAD samples were divided into high-risk and low-risk groups on the basis of the risk score's median. According to Kaplan Meier curve analysis, samples from high-risk groups had worse outcomes than those from low-risk groups. The time-dependent receiver operating characteristics (ROC) analysis revealed that the risk model could predict the prognosis of PAAD accurately. A nomogram accompanied by calibration curves was presented for predicting 1-, 2-, and 3-year survival in PAAD patients. More importantly, 4 small molecular compounds (A.443654, PD.173074, Epothilone. B, Lapatinib) were identified, which might be potential drugs for the treatment of PAAD patients. Finally, the depletion of GSDMC inhibits the proliferation, invasion, and migration of pancreatic adenocarcinoma cells.

**Conclusion:**

In this study, we developed a pyroptosis-related prognostic model based on IL18, CASP4, NLRP1, NLRP2, and GSDMC , which may be helpful for clinicians to make clinical decisions for PAAD patients and provide valuable insights for individualized treatment. Our result suggest that GSDMC may promote the proliferation and migration of PAAD cell lines. These findings may provide new insights into the roles of pyroptosis-related genes in PAAD, and offer  new therapeutic targets for the treatment of PAAD.

**Supplementary Information:**

The online version contains supplementary material available at 10.1186/s12967-022-03632-z.

## Introduction

PAAD is a cancerous tumor characterized by rapid spread and poor prognosis, resulting in 466,003 new deaths in 2020 and 495,773 new cases of pancreatic cancer worldwide [1]. Studies showed that pancreatic cancer incidence and mortality rate are nearly equivalent and the 5-year survival rate is estimated at only 1% [2]. The majority of PAAD patients are already suffering from advanced diseases when they are diagnosed. However, up to 80% of pancreatic cancers cannot be resected because of their highly malignant and early metastasis [3]. Patients with pancreatic cancer after resection  still have a poor prognosis [4]. Hence, it is urgent to develop a prognostic model and identify biomarkers in  diagnosis of PAAD.

Pyroptosis is a type of regulated necrotic cell death induced by inflammatory caspases. It mainly relies on the inflammation to activate a part of the caspase family of proteins, so that inflammation cleaves the Gasdermin protein, activates the Gasdermin protein, and the activated Gasdermin protein translocates to the cell membrane to form holes, then the cell swells, the cell membrane ruptures, and finally leads to the efflux of the cytoplasm and the formation of pyroptosis. Caspase-1, 4, 5, 11 are pro-inflammatory cysteine proteases, all belong to the cysteine aspartate proteolytic enzyme family, and this class of proteases is critically involved in the body's generation of inflammatory responses and innate immune responses [5]. Caspase-3 is a master regulator of apoptosis, while those associated with inflammation include caspase-1, 4, 5, 11, 12, 13, 14, among which caspase-1, 4, 5, 11 mainly mediates pyroptosis [6]. Endogenous and exogenous stimulatory signals act on the inflammasome through different pathways to activate caspase-1, which mediates the osmotic swelling cleft of the cell, the formation of small pores in the cell membrane, the efflux of intracellular substances (such as lactate dehydrogenase, etc.). IL-1 β and IL-18 precursor cleaves and induces the synthesis and release of other inflammatory factors, adhesion molecules. Amplifying the local and systemic inflammatory response is the main mechanism by which pyroptosis occurs [7]. It has been shown that pyroptosis plays a dual role in promoting and inhibiting the development of cervical cancer. Studies have shown that the NLRP3 inflammasome is involved in the innate immune response to cervical cancer, and its expression is widely present in tumor cells [8]. NLRP3 inflammatory activation can be achieved through humans, lysosomal rupture, and reactive oxygen species. In cervical cancer, the NLRP3 inflammasome is mainly activated by reactive oxygen species to induce pyroptosis [9]. In HPV infected cervical cancer cells, aim2 can play a tumor suppressive role by stimulating pyroptosis [10]. However, several studies have found that removal of pro-inflammatory factors produced by pyroptosis can inhibit cervical cancer cell growth while impairing the body’s immune effect on tumor cells [11, 12].

An increasing number of studies have shown that pyroptosis plays an important role in cancer progression. Therefore, in-depth study of the role of pyroptosis in pancreatic carcinogenesis and progression, as well as the establishment of a relevant prognostic model of pyroptosis, is of great importance for the treatment of PAAD. To the best of our knowledge, there is no pyroptosis-related prognostic model in PAAD has been established to predict the prognosis of patients with PAAD. Therefore, a novel prognostic model based on pyroptosis-related genes for predicting survival of patients with PAAD is highly needed. In this present study, we aimed to establish a prognostic model on the basis of pyroptosis-related genes to  predict the prognosis of patients with PAAD. In addition, we verified the function of GSDMC in PANC-1  and CFPAC-1 cells, which might be a promising therapeutic target for the treatment of PAAD. Our study systematically explored the prognostic value of pyroptosis-related genes and their correlations with clinical characteristics, thus shedding light on the promising roles of pyroptosis-related genes as potential prognostic biomarkers and novel therapeutic targets for PAAD patients.   

## Materials and methods

### Data acquisition and preprocessing

The RNA sequencing (RNA-seq) data of 178 PAAD samples and 167 normal pancreatic samples with their clinicopathological parameters were downloaded from the cancer genome atlas (TCGA, https://portal.gdc.cancer.gov) and Genotype-Tissue Expression (GTEx, https://www.gtexportal.org/home/) databases. The RNA-seq data and clinicopathological features of 186 PAAD samples were downloaded from Gene Expression Omnibus GSE71729 and GSE57495 datasets (https://www.ncbi.nlm.nih.gov/geo/). Gene expression data were normalized by the “Sanger box” tools before further analysis (http://sangerbox.com/).

### Data normalization

In order to integrate the expression data from TCGA and GEO database (GSE71729 and GSE57495), we performed the batch normalization to remove batch effects. Firstly, we downloaded mRNA-seq FPKM data of TCGA PAAD patients. Secondly, we downloaded the microarray expression data from GEO database (GSE71729 and GSE57495). Thirdly, these expression data from TCGA and GEO database were log2-transformed. Finally, batch normalization was performed across abovementioned data using the combat function in "sva" package in R software (version 4.1.2) [13]. This method was widely utilized to combine different datasets in previous studies [14–16].

### Identification of differentially expressed pyroptosis-related genes

The “DESeq2” package was used to identify differentially expressed pyroptosis-related genes in 178 PAAD samples and 167 normal samples. P < 0.05 and |log2 fold change (FC)|> 1.2 were set as as cut-off values. The volcano of pyroptosis-related genes and heatmap of differentially expressed pyroptosis-related genes were drawn using the OmicStudio tools (https://www.omicstudio.cn/tool). Protein protein interactions (PPIs) were plotted by using string database (https://string-db.org/) and boxplots were drawn with the R package “ggpubr”. The minimum interaction score required for PPI analysis was set at 0.4 (medium confidence).

### Enrichment analysis of differentially expressed pyroptosis-related genes

The biological process enrichment of 25 differentially expressed genes were analyzed with Gene Ontology (GO) and Kyoto Encyclopedia of Genes and Genomes (KEGG) through R statistical software including “clusterProfiler”, “org.Hs.eg.db”, “enrichplot”, “ggplot2”, and “GOplot” packages. In addition, gene set enrichment analysis (GSEA) was performed in order to identify the biological process and signaling pathways that differ between high-risk groups and low-risk groups in PAAD. Our reference gene sets were derived from the C2 subcollection (c2.cp.kegg.v7.5.1.symbols.gmt). The significance thresholds were determined by 1000 permutation analyses, and we considered significant results when the p value was less than 0.05.

### Identification of prognostic genes

Our training set consisted of 178 PAAD samples and 167 normal pancreatic samples from the TCGA and GTEx databases. To investigate the relationship between the expression levels of pyroptosis-related genes and overall survival (OS) of PAAD patients, we conducted a univariate Cox regression analysis using the "survival" package. A significant filtering criterion was set at p < 0.05 for further analysis. We next eliminated gene collinearity and reduced the number of genes using LASSO Cox regression. Finally, we conducted multivariate Cox regression analysis on the basis of univariate Cox regression.

### Construction and validation of a prognostic model based on pyroptosis-related genes

The risk score was calculated according to the centralized and standardized PAAD mRNA expression data in the train set.

$${\text{Risk}}\, {\text{score}} = \sum\nolimits_i^n {{x_i}{y_i}}$$

X represents the coefficient of pyroptosis-related genes in LASSO Cox regression analysis, Y represents the gene expression of pyroptosis-related genes. PAAD patients were divided into high-risk and low-risk groups based on the median risk score, and the overall survival (OS) between these two groups was analyzed. Receiver operating characteristic (ROC) curves were produced by the timeROC package to evaluate the prognostic efficiency of the model. To make the model more convincing, we utilized the PAAD cohort in the GEO database for validation. The expression of each pyroptosis-related genes was also normalized, and the risk score was then calculated by the above formula. PAAD patients in the GEO cohort were also grouped into high-risk and low-risk groups according to the median risk score, and the OS between the two groups was also compared. Next, to determine if risk score was an independent prognostic factor for OS in PAAD patients in the train set, univariate and multivariate Cox regression analyses were conducted. Covariates included age, gender, grade, stage, T, and N.

### Construction of nomogram and calibration curves

The nomogram was built using the “RMS” package of R software to predict individual survival probability, and calibration curves for the prediction of 1 -, 2-, and 3-year survival rate of PAAD patients were plotted.

### Drug sensitivity analysis

Using the pRRophetic package in R software, the sensitivity score of each small moleular compound was calculated for each patient in the high-risk group and low-risk group. Then, we used PubChem (https://pubchem.ncbi.nlm.nih.gov/) website to visualize the conformations of drugs in 3D.

### Cell culture

CFPAC-1 and PANC-1 cells were purchased from Procell Life Science and Technology Co., Ltd. (Wuhan, China). CFPAC-1 and PANC-1 cells were cultivated in RPMI-1640 (Hyclone) supplemented containing 2 mM L-glutamine and 10% FBS (Life Technologies).

### siRNA sequence

The siRNA sequences were as follows: si-GSDMC-1:5′-GGAUCCAGAGCC AUCAUUU-3′. si-GSDMC-2: 5′- CCUAGA AACUGUUGUGACA-3′.

### CCK8 assay

Cell viability was determined by CCK8 kit. In short, CFPAC-1 and PANC-1 cells transfected with si-GSDMC-1 or si-GSDMC-2 were inoculated in 96 well plates × 1000 cells/well). Add CCK8 and use the multimode microplate reader at 0, 24, 48, 72 h respectively. The optical density of each well was measured at 450 nm. Each experiment was repeated three times.

### EdU assay

EdU kit was used for EdU determination (Ribobio, # C10310-2). The EdU test solution was inoculated into CFPAC-1 and PANC-1 cells transferred with si-gsdm-1 or si-gsdm-2, respectively. Continue to culture in the incubator for 2 h, and then fix with 4% paraformaldehyde for 30 min. The dye is then dyed according to the manufacturer's scheme and the image is taken using EVOS FL automatic microscope. Finally, using Image J software to count the number of cells. Each experiment was repeated three times.

### Wound healing assay

The ability of cell migration was evaluated by a wound healing experiment. CFPAC-1 and PANC-1 cells transfected with si-GSDMC-1 or si-GSDMC-2 were inoculated in 6-well plates. When the cells reach reaching a confluence of 100%. Use a 10 µl pipette to form a wound in the center of the cell monolayer, and then continue to culture in the incubator. At a specific time, using Image J software to count the wound area. Each experiment was repeated three times.

### Western blotting

 Two small inferring RNAs (siRNAs) were employed to knock down GSDMC. After 48 h of transfection, cells were lysed in RIPA buffer with a phosphatase inhibitor cocktail (biomake, #B14001, #B15001). Proteins were loaded and separated by electrophoresis on SDS–polyacrylamide gel electrophoresis (SDS-PAGE) and transferred to a nitrocellulose membrane. The signals were visualized using the ECL Kit (Meilunbio, #MA0186). Antibodies used were anti-GSDMC (diluted 1:1000, proteintech, #27,630-1AP) and anti-ACTIN antibody (diluted 1:1000, Abclonal, #AC026).

### Lentiviral production, infection, and construction of stable cell lines

We constructed shRNA sequences (sh-1:5′-GGAUCCAGAGCC AUCAUUU-3′. sh-2:5′-CCUAGA AACUGUUGUGACA-3′) using the plko.1-puro-gfp vector, and a scramble sequence (5′-TTCTCCGAACGTGTCACGT-3′) was designed as a negative control. We also constructed GSDMC-overexpressing lentivirus using the pCDH-CMV-Puro vector (GeneChem, China). Next, we followed the manufacturer's instructions for lentiviral transfection. Briefly, shRNA or pCDH plasmid was cotransfected with the packaging plasmids psPAX2 and MD2G in 293 T cells using lipofectamine 3000. Supernatants were collected 48 h after transfection and filtered through 0.22 μm low protein binding filters. 1 ml of supernatant were used to transduce PANC-1 or CFPAC-1 cells, and the medium was changed 2 days after infection. The infected cells were screened by puromycin for 10 days.

### Cell cycle analysis

Propidium iodide (PI) staining was utilized to analyze cell cycle. In short, for the cell cycle analysis, cells (1 × 10^7^) were washed using PBS and fixed using 70% ethanol for 30 min at room temperature. We then stained the cells with PI containing RNase A (Thermo, # F10797) after washing them three times with PBS. A FACScan (Millipore) was used to measure the red signal and ModFIT LT v3.1 software was used to analyze the FSC data.

### Chemical treatment of cells

In six-well dishes at a density of 1000 cells per well, the PANC-1 cells were trypsinized and plated. Cells were allowed to attach overnight and then exposed to corresponding concentrations of chemical treatment. Approximately 48 h after chemical treatment, we replaced the media with fresh media, and incubated the plates at 37 °C.

### Statistical analysis

Statistical analysis was performed by two-tailed unpaired t-test, one-way ANOVA and two-way ANOVA in GraphPad Prism software (version 8.0.2). When the p value was less than 0.05, the results were considered statistically significant.

## Results

### Identification of differentially expressed pyroptosis-related genes in PAAD

**Fig. 1:**
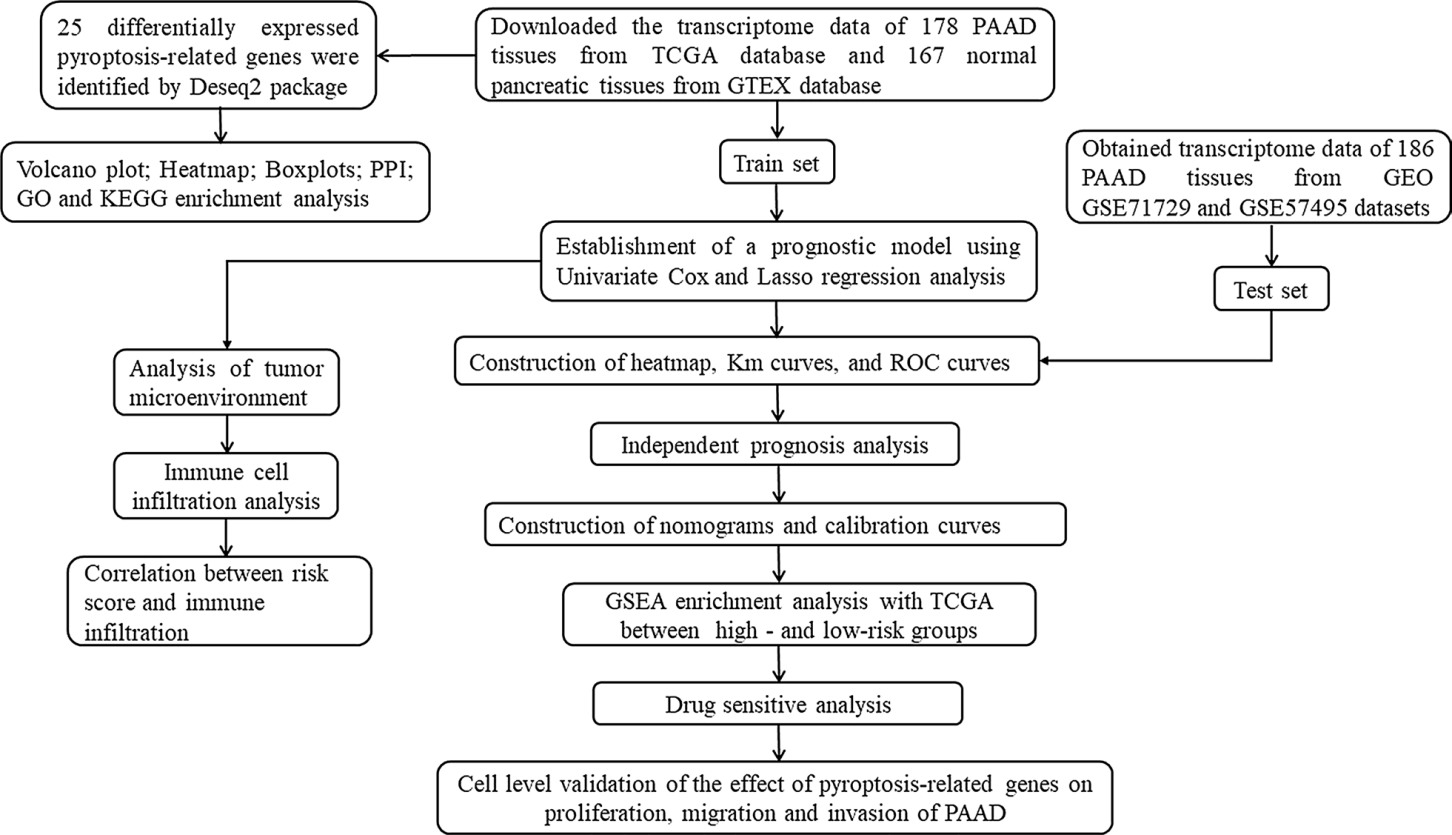
The flowchart of our research process

**Fig. 2:**
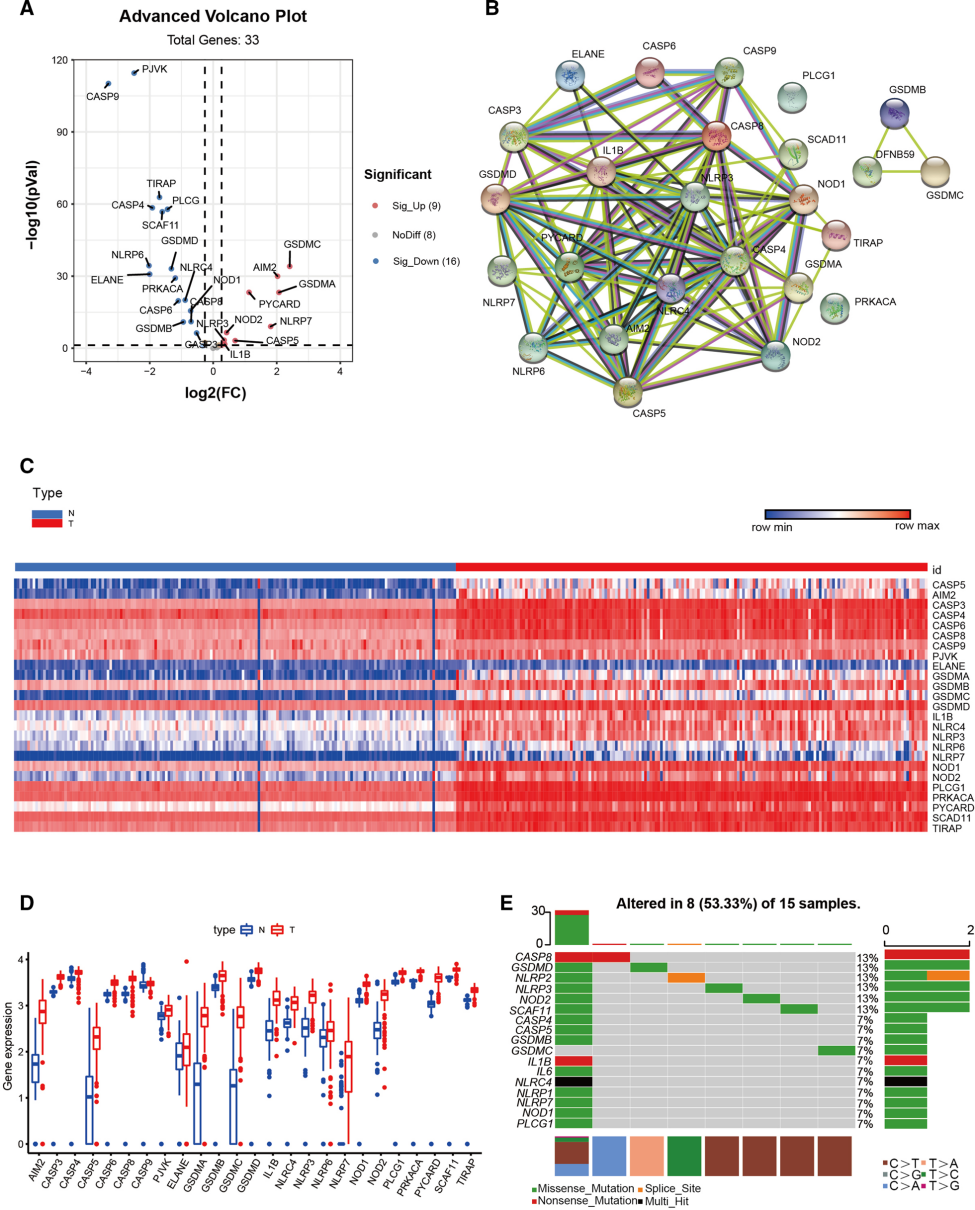
Differentially expressed pyroptosis-related genes between PAAD tissues and normal tissues. **A** Volcano plot indicates pyroptosis-related genes, with red dots indicating high expression and blue dots indicating low expression. **B** The protein–protein interaction (PPI) network shows the interaction of pyroptosis-related genes (interaction score = 0.4). **C** Heatmap of differentially expressed pyroptosis-related genes, with red indicating high expression, blue indicating low expression, n representing normal tissues, and t representing tumor tissues. **D** Boxplots of differentially expressed pyroptosis-related genes, with red boxes representing tumor groups and blue boxes representing normal groups. **E** Mutation analysis of differentially expressed pyroptosis-related genes in TCGA cohort

The detailed workflow of our study is shown in (Fig. 1). We obtained 178 PAAD patients from TCGA and 167 normal tissues from GTEx. A total of 25 differntially expressed pyroptosis-related genes were identified based on the cutoff criteria of |log2 (fold change) |> 1.2 and false discovery rate (FDR) < 0.05 from 33 pyroptosis-related genes using R package “DESeq2”. In PAAD patients, volcano plots, heatmaps, and boxplots demonstrated that 16 pyroptosis-related genes were significantly downregulated, whereas 9 pyroptosis-related genes were upregulated (Fig. 2A, C, D). The protein-protein interaction network of these differentially expressed pyroptosis-related genes was shown in (Fig. 2B). Moreover, many mutations in patients with PAAD have been observed in these differentially expressed pyroptosis-related genes (Fig. 2E).

### Functional enrichment analysis

GO and KEGG pathway enrichment analyses were performed in order to better understand the functions of differentially expressed pyroptosis-related genes. The analysis of GO enrichment revealed that these differentially expressed pyroptosis-related genes were primarily associated with the formal regulation of cytokine production and defense responses to bacteria (Additional file 1: Fig. S1A). In addition, the analysis of KEGG revealed that these differentially expressed pyroptosis-related genes were involved in platinum drug resistance, apoptosis-multiple species, ERbB signaling pathway, and apoptosis (Additional file 1: Fig. S1B). This suggests that these pyroptosis-related genes are involved in other biological processes besides pyroptosis.

### Construction of a prognostic model based on pyroptosis-related genes in the train set

**Fig. 3:**
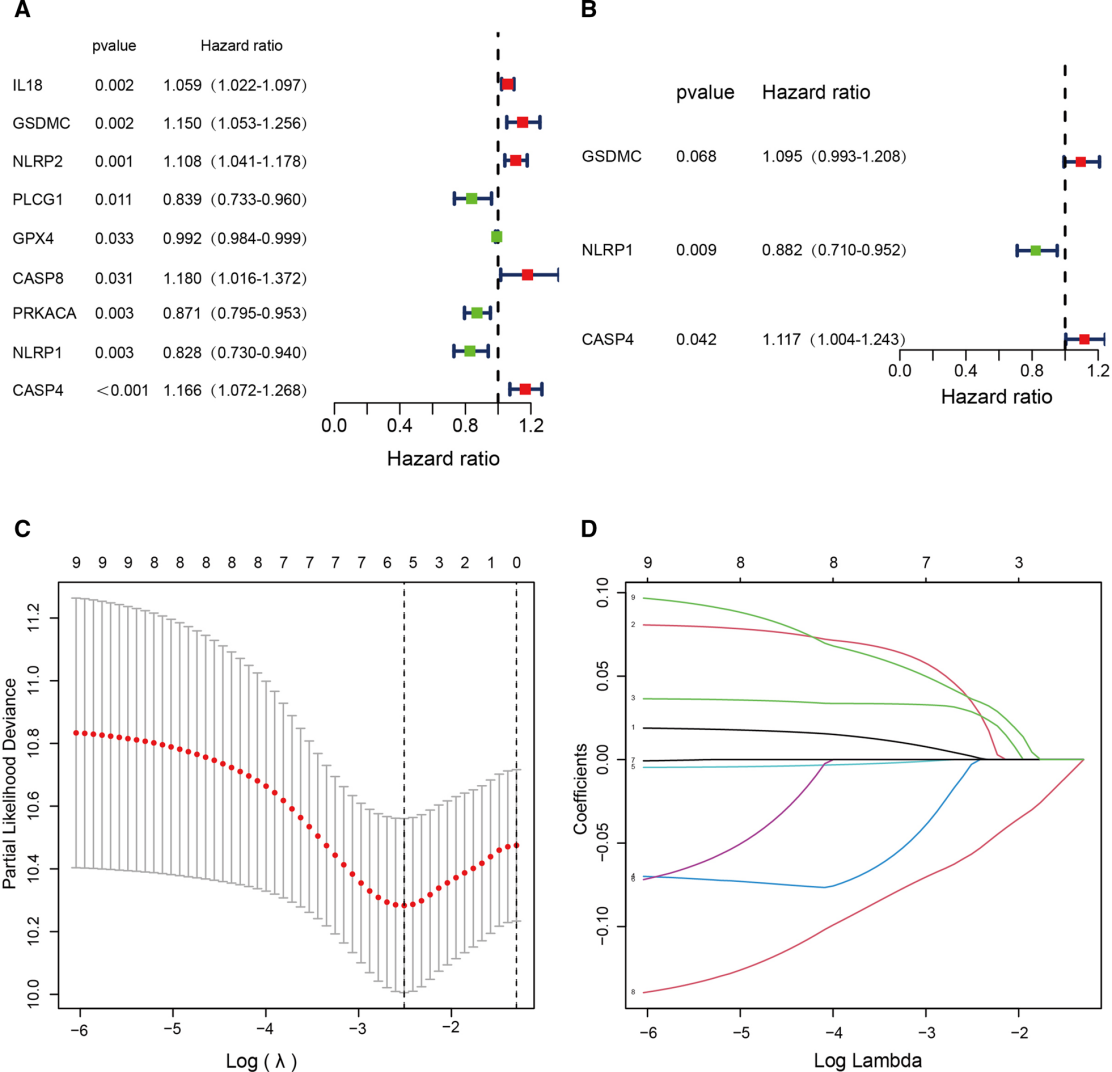
Construction of a risk prognostic model based on pyroptosis-related genes in the TCGA cohort. **A** Univariate Cox regression analysis was performed for all pyroptosis-related genes. A value of P < 0.05 was considerated statistically significant. **B** Multivariate Cox regression analysis was performed on the genes derived from the univariate Cox regression analysis. **C **LASSO regression of the 5 OS-related genes. **D** Cross-validation for tuning the parameter selection in the LASSO regression

As shown in Fig. 3A, we screened out 9 pyroptosis-related genes with P < 0.05 using univariate Cox regression analysis, including five potential risky genes (IL18, GSDMC, NLRP2, CASP8, and CASP4) and four potential protective genes (PLCG1, GPX4, PRKACA, and NLRP1). On the basis of the univariate Cox regression, we then performed LASSO regression analysis (Fig. 3C, D). Next, we constructed the prognostic pyroptosis-related model using IL18, CASP4, NLRP1, NLRP2, and GSDMC by LASSO regression. Finally, we performed multivariate Cox regression analysis and identified three pyroptosis-related genes, two of which were potential risk genes and one of which was potential protective genes (Fig. 3B).

**Fig. 4:**
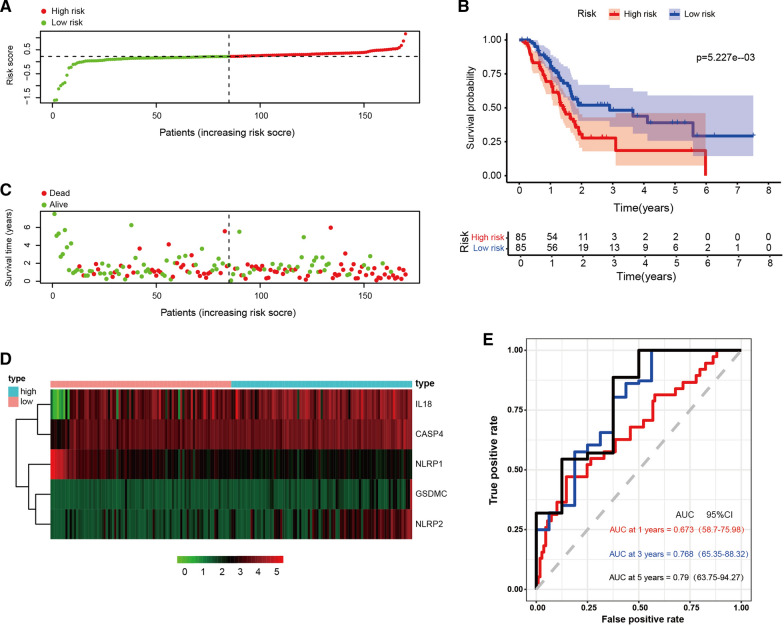
Construction of risk model in TCGA cohort. **A** The patients were equally divided into two groups according to the threshold of the median risk score. Green represents the low-risk group. Red represents the high-risk group. **B** Kaplan Meier curves showing the overall survival of patients in the high-risk and low-risk groups. **C** Survival status of patients with PAAD in high and low risk groups. Green represents survival. Red represents death. **D** Heatmap showing the expression of the five pyroptosis-related genes. Pink represents the low-risk group. Bright blue represents the high-risk group. **E** The predictive efficiency of the risk score was verified by the ROC curve

We constructed a prognostic index for all cancer samples calculated by the formula: Risk score = expression level of IL18 × 0.002067 + expression level of GSDMC × 0.034755 + expression level of NLRP2 × 0.028693 + expression level of PLCG1 × (− 0.00285) + expression level of NLRP1 × (− 0.05665) + expression level of CASP4 × 0.036385. In order to confirm whether this pyroptosis-related model could predict the prognosis of patients with PAAD, we divided the 170 patients into a high-risk group (n = 85) and a low-risk group (n = 85) according to the threshold of median risk score (Fig. 4A). The high-risk group had a higher mortality rate and shorter survival time compared with the low-risk group. Higher scores were associated with a worse prognosis for PAAD patients (Fig. 4C). IL18, CASP4, GSDMC, and NLRP2 were highly expressed in the high-risk group, and NLRP1 was lowly expressed in the high-risk group (Fig. 4D). Kaplan Meier curves showed that patients in the high-risk group had a worse prognosis (P < 0.05, Fig. 4B). Time dependent ROC analysis showed that the prognostic accuracy of OS was 0.673 at 1 year (95% CI 58.7–75.98), 0.768 at 3 years (95% CI 65.35–88.32), and 0.790 at 5 years (95% CI 63.75–94.27) (Fig. 4E). The results of these studies suggested that the pyroptosis-related gene signature in our model could be helpful for predicting PAAD prognosis, and the model we established has excellent accuracy of predicting prognosis in the training set.

### Validation of the prognostic model in the test set

To verify the accuracy of the established prognostic model, we obtained 186 pancreatic cancer patients from GEO and calculated the risk score using the same formula in the training set. According to the median value of the risk score, 99 patients in the GEO cohort were classified into the low-risk group and 87 patients into the high-risk group (Additional file 2: Fig. S2A). Patients in the low-risk group had longer survival time than those in the high-risk group (Additional file 2: Fig. S2C). IL18, CASP4, GSDMC, and NLRP2 were highly expressed in the high-risk group and NLRP1 was lowly expressed in the high-risk group (Additional file 2: Fig. S2D). In addition, Kaplan Meier analysis also indicated that low-risk group and high-risk group had significantly different survival rates (P < 0.05, Additional file 2: Fig. S2B), which was consistent with the result in training set. Time dependent ROC analysis showed that the prognostic accuracy of OS was 0.590 at 1 year (95% CI 50.2–67.33), 0.554 at 3 years (95% CI 44.62–66.33), and 0.658 at 5 years (95% CI 57.75–73.94) (Additional file 2: Fig. S2E). As a result, it was clear that the model we established has acceptable accuracy of predicting prognosis in the test set.

### Independent prognosis analysis of Riskscore and Clinical Characterstics

To verify whether the risk score and clinical characteristics could act as independent prognostic factors, we performed univariate and multivariate independent prognosis analyses. The results of univariate independent prognosis analysis showed that age, N stage, and risk score were significantly correlated with the OS of PAAD patients (Additional file 3: Fig. S3A). Multivariate independent prognosis analysis showed that the risk score could be an independent predictor (p < 0.001) (Additional file 3: Fig. S3B). Moreover, we evaluated the relationship between the expression of pyroptosis-related genes and clinical features. Results showed that NLRP1 was negatively correlated with grade (Additional file 4: Fig. S4A, P = 0.022), N (Additional file 4: Fig. S4B, P = 0.046), T stage (Additional file 4: Fig. S4C, P = 0.046). NLRP2 was positively correlated with grade (Additional file 4: Fig. S4D, P = 0.022). GSDMC was negatively correlated with stage (Additional file 4: Fig. S4E, P = 0.010). CASP4 was positively correlated with grade (Additional file 4: Fig. S4F, P = 0.012). The risk score was positively correlated with grade (Additional file 4: Fig. S4G, P = 5.002e − 04). Based on the results of these studies, we concluded that our model could be a reliable prognostic biomarker.

### Construction of nomogram and calibration curves

**Fig. 5:**
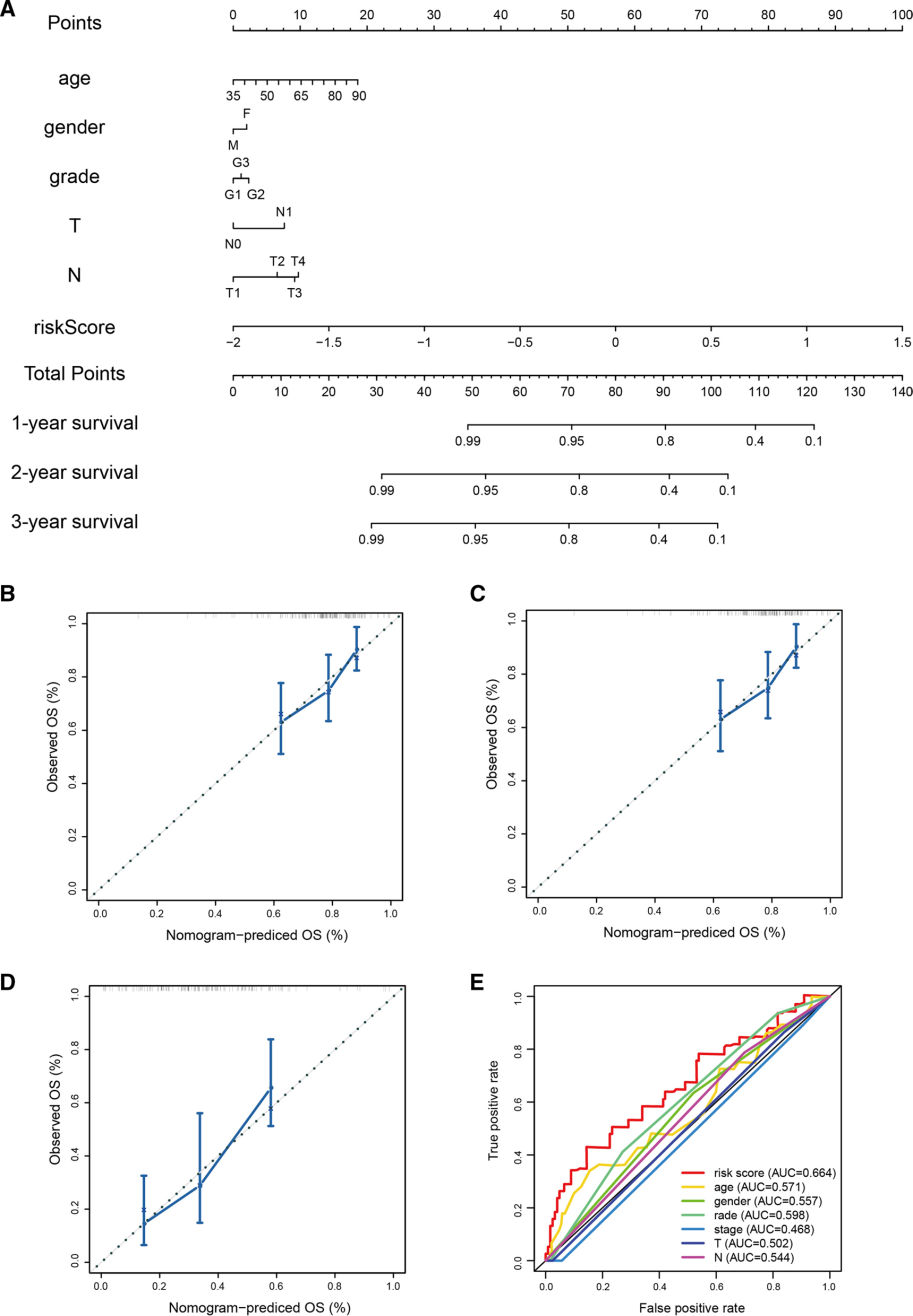
Nomogram to predict survival probability of pancreatic cancer patients. **A** Nomogram combining risk score with pathologic features. **B**–**D** Calibration plots for predicting 1 -, 2 -, 3-year OS of patients. **D** ROC curves for prediction of survival by the risk score and other variables (age, gender, stage, N stage, T stage)

In order to provide clinicians with a better quantitative method to forecast the prognosis of the patients with PAAD, we established a nomogram combining age, gender, N, T, and risk scores. The nomogram showed that the risk score was an important factor among various clinical parameters (Fig. 5A). Furthermore, we constructed calibration curves, which showed that the nomogram had a good match with the actual survival of patients with PAAD (Fig. 5B–D). Compared with traditional prognostic scoring systems, our model had a higher AUC value (AUC = 0.664, Fig. 5E). Based on these findings, we found that the nomogram containing our risk scores can be used to accurately predict the OS of PAAD patients.

### GSEA enrichment analysis

We performed GSEA enrichment analysis between the high-risk and low-risk groups in the training set and the validation set, respectively. In the training set, the results showed that the enriched pathways in the high-risk group included apoptosis, tight junction, spliceosome, axon guidance, and natural killer cell-mediated cytotoxicity. The main enriched pathways in the low-risk group were neuroactive ligand receptor interaction, taste transduction, autoimmune thyroid disease, chemokine signaling pathway, focal adhesion (Additional file 5: Fig. S5A). In the validation set, the results indicated that the main enriched pathways included proteasome, glycosylphosphatidylinositol GPI anchor biosynthesis, nucleotide excision repair, aminoacyl tRNA biosynthesis, and DNA replication in the high-risk group, while in the low-risk group, the main enriched pathways were cardiac muscle contraction, long term depression, vascular smooth muscle contraction, calcium signaling pathway, and amyotrophic lateral sclerosis ALS (Additional file 5: Fig. S5B).

### Evaluating the Therapeutic Response in the high-risk and low-risk group

**Fig. 6:**
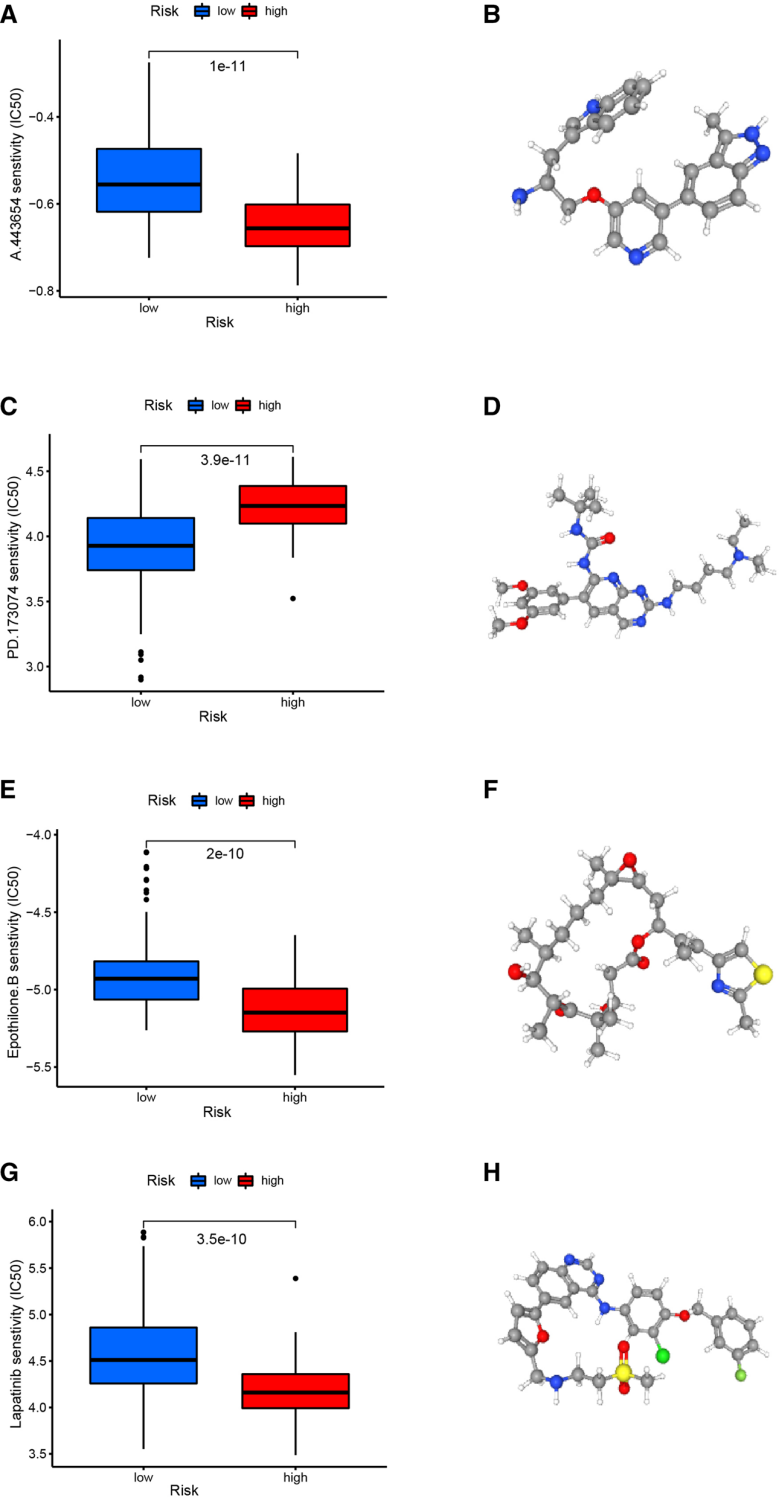
The screened drugs for PAAD treatment. IC 50 value of A.443654 (**A**), PD 173,074 (**C**), Epothilone. B (**E**), Lapatinib (**G**) in high-and low-risk patients with PAAD. The corresponding 3D structures are shown in (**B**), (**D**), (**F**) and (**H**), respectively

To predict the response to chemotherapy, we used the pRRophetic algorithm to estimate the chemotherapeutic response based on the half-maximal inhibitory concentration (IC50) available in the genomics of drug sensitivity in cancer (GDSC) database for patients with PAAD. A total of 49 small molecular compounds with significantly different responses were identified between high- and low-risk groups in our study (Additional file 11: Table S1). The top four small molecular compounds were found to have the lowest P values between the high-risk and low-risk groups, including A.443654 (P = 1e−11, Fig. 6A), PD.173074 (P = 3.9e−11, Fig. 6C), Epothilone.B (P = 2e−10, Fig. 6E), Lapatinib (P = 3.5e−10, Fig. 6G). PAAD patients in the high-risk group were more sensitive to A.443654, Epothilone.B, and Lapatinib, while those in the low-risk group were more sensitive to PD.173074. 3D conformations of these 4 small molecular compounds were then visualized through PubChem website (Fig. 6B, D, F, H). In light of these findings, these small molecular compounds might be potential PAAD treatment agents, but further analysis is needed in the near future. Our results provide potential molecular chemotherapy compounds for patients with PAAD.

### Analysis of Tumor Microenvironment and Immune cell infiltration

**Fig. 7:**
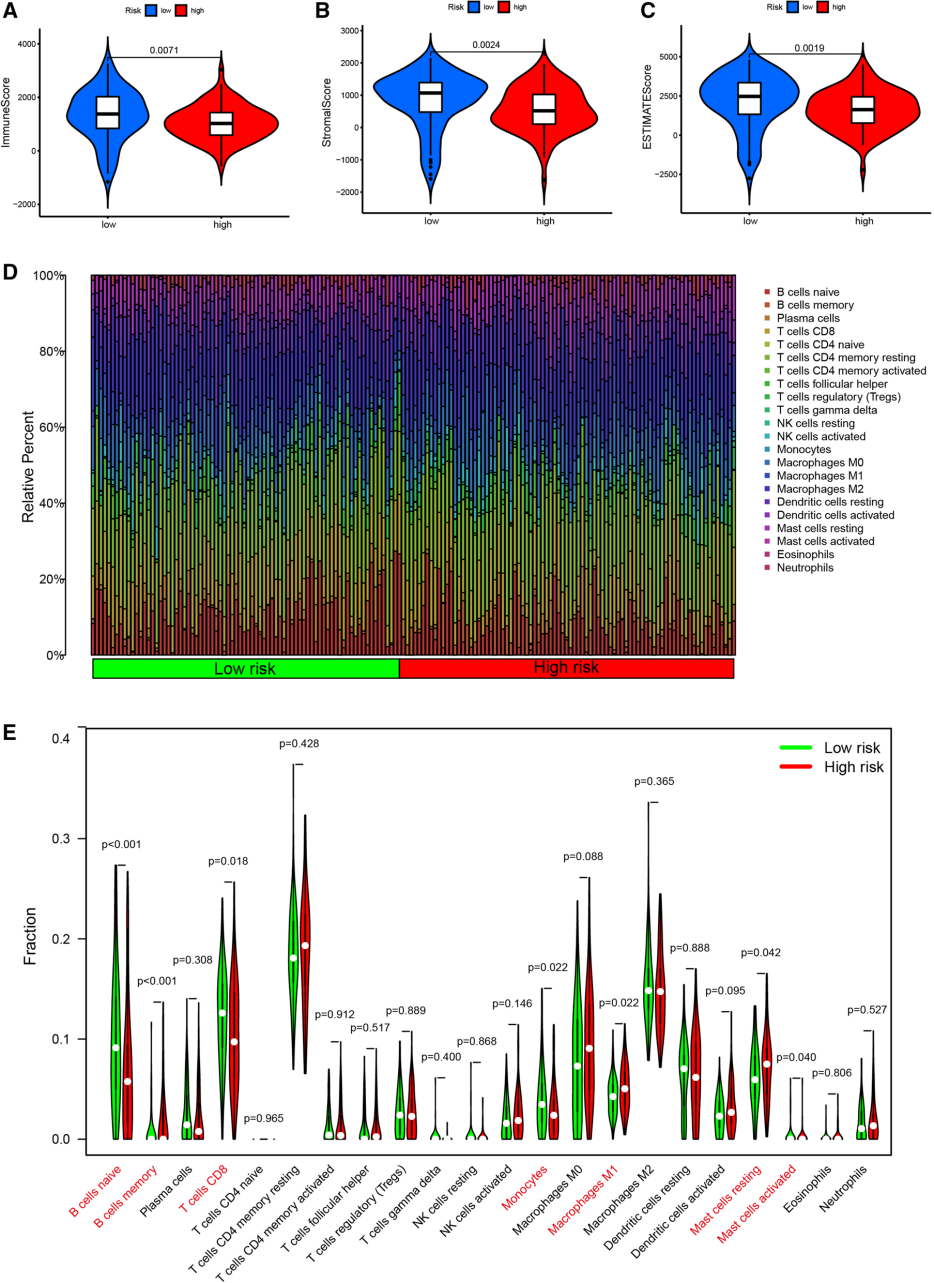
Tumor Microenvironment and immune cell infiltration analysis. Violin plots represent the relationship of risk score with immune score (**A**), stromal score (**B**) and ESTIMATE score (**C**). (**D**) Relative proportion of immune cell infiltration in high-risk and low-risk group. Green represents the low-risk group. Red represents the high-risk group

The tumor microenvironment, including immune cells and stromal cells, is believed to play an essential role in tumor development, metastasis, recurrence, and drug resistance. Therefore, we combined immune score, stromal score, and ESTIMATE score to analyze the association of these scores with the risk score. The results showed risk score was significantly correlated with immune score (P = 0.0071), stromal score (P = 0.0024) and ESTIMATE score (P = 0.0019, Fig. 7A–C). The proportion of 22 subtypes of immune cells in PAAD patients was calculated using the CIBERSORT algorithm and a threshold of P-value < 0.05 was considered as cut-off criteria (Fig. 7D). The infiltration levels of B cell memory, macrophages M1, and mast cell resting in the high-risk group were higher than those in the low-risk group. However, the infiltration levels of B cells Naive, T cells CD8, monocytes, and mast cells activated in the low-risk group were higher than those in the high-risk group (Fig. 7E, Additional file 6: Fig. S6A). In addition, we evaluated the correlation between the risk score and five immune cells. The results indicated that T cells CD8 (R = − 0.17, P = 0.027), monocytes (R = − 0.18, P = 0.023), and B cells Naive (R = − 0.27, P = 0.00047) were positively correlated with risk score, while macrophages M1 (R = 0.19, P = 0.016), and B cells memory (R = 0.25, P = 0.0011) were negatively correlated with risk score (Additional file 6: Fig. S6B-F).

### Kaplan-Meier plots of Prognostic Genes

We plotted Kaplan-Meier plots to test whether the expression of  prognostic pyroptosis-related genes was correlated with the prognosis of PAAD. The results show that PAAD patients with high expression of NLRP1 had a better prognosis, while PAAD patients with low expression of NLRP2, GSDMC, IL18 and CASP4 had a better prognosis (Additional file 7: Fig.s S7A-E).

### Knockdown of GSDMC inhibited PAAD cell proliferation and migration

**Fig. 8:**
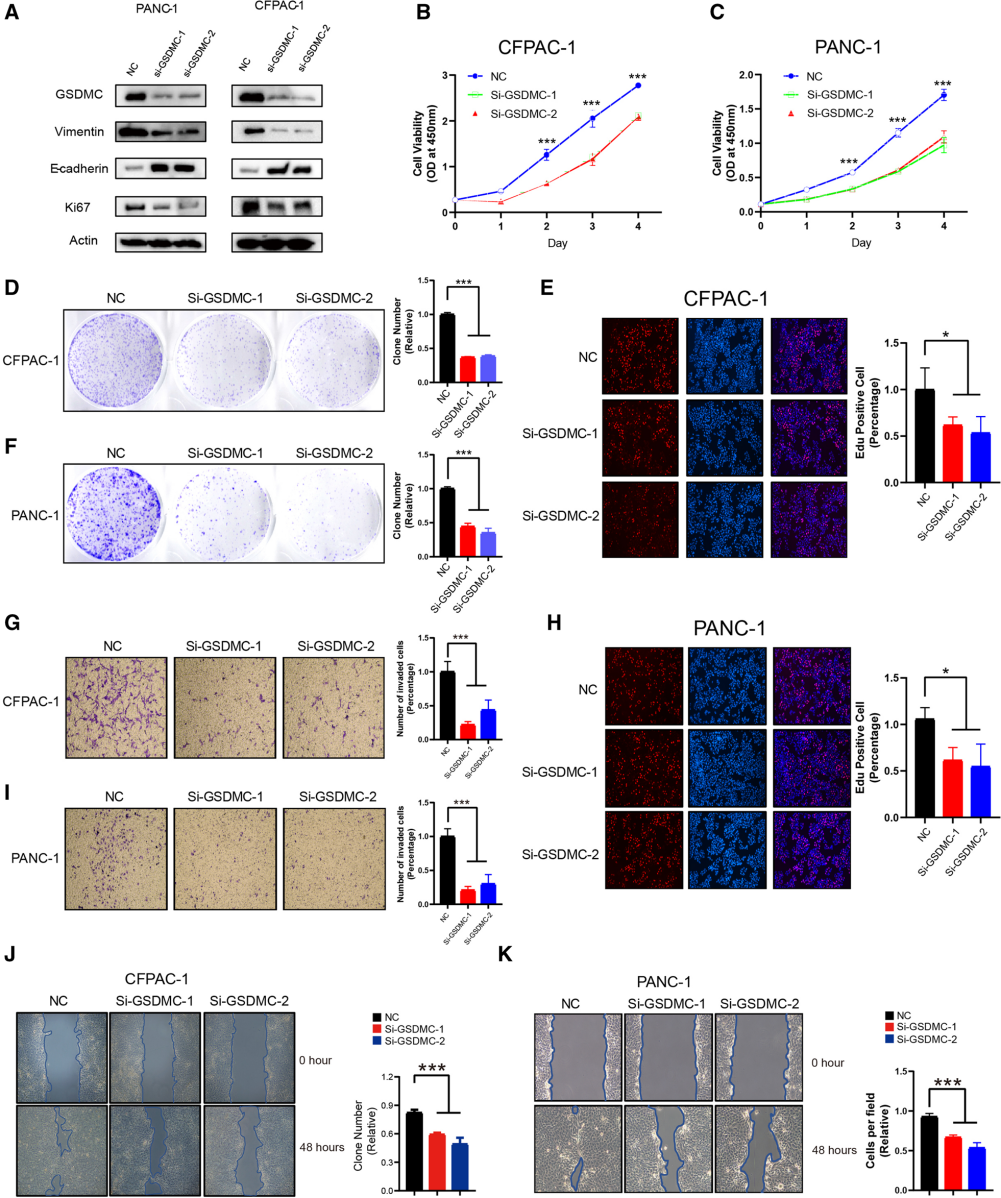
Knockdown of GSDMC inhibited PAAD cell proliferation and migration. **A** Western blot results showing the expression levels of indicated proteins in PANC-1 (left panel) or CFPAC-1 (right panel) cellline transfected with scrambled or two independent siRNA targeting GSDMC, respectively. **B**, **C** Cell viability was determined by CCK8 assay in the PANC-1 (B) and CFPAC-1 (C) cell lines transfected with either scrambled or two independent GSDMC siRNA targeting GSDMC, respectively. ***p < 0.001 by one-way ANOVA. **D**, **F** Colony formation assay of CFPAC-1 (D) and PANC-1 (F) cell lines with either scrambled or two independent siRNA targeting GSDMC, respectively. ***p < 0.001 by one-way ANOVA. **E**, **H** Edu assay to show the cell proliferation of PANC-1 (E) and CFPAC-1 (H) cell lines transfected with either scrambled or two independent siRNA targeting GSDMC, respectively. *p < 0.05 by one-way ANOVA. **J**, **K** Wound healing assay of PANC-1 (J) and CFPAC-1 (K) cell migration capability following transfected with scrambled or two independent siRNA targeting GSDMC, respectively. ***p < 0.001 by one-way ANOVA

To investigate the role of GSDMC in PAAD cells, two siRNAs (GSDMC-1, GSDMC-2) were designed to silence GSDMC expression in PANC-1 and CFPAC-1 cells. GSDMC expression was verified by western blot, and abovementioned two siRNAs have been found to be effective to knock down the expression of GSDMC. The western blotting demonstrated that knockdown of GSDMC by siRNA in PANC-1 and CFPAC-1 cells reduced vimentin and Ki-67, but increased E-cadherin expression (Fig. 8A). According to these results, GSDMC could promote cell proliferation and invasion in PAAD cells. To investigate the effects of low expression GSDMC on cell proliferation, invasion, migration. CCK8, colony formation assays, EdU, Transwell and wound healing experiments were performed on CFPAC-1 and PANC-1 cells transfected with Si-GSDMC-1 or Si-GSDMC-2, respectively. The CCK8 results showed that the proliferation ability of CFPAC-1 cells in the NC group was significantly stronger than that in the other groups at 24, 48, 72 and 96 h (P < 0.05, Fig. 8B). By tumor cell colony formation assay, we found that the proliferation ability of CFPAC-1 cells transfected with Si-GSDMC-1 or Si-GSDMC-2 was significantly lower than that of NC group (Fig. 8D). The results of EdU staining assay showed that the effect of knocking down the GSDMC gene on the proliferation of CFPAC-1 cells was significant, and the ability of CFPAC-1 cells transfected with Si-GSDMC-1 or Si-GSDMC-2 to proliferate was significantly lower than that of the NC group (Fig. 8E). The results of transwell assay showed the absence of GSDMC, limiting the migration and invasion of CFPAC-1 cells. The results of the wound healing experiment showed a lower migratory ability of the low expressing CFPAC-1 cells. The results were similar to CFPAC-1 in the PANC-1 cell line (Fig. 8C, F, H, I, K). These results indicated that knockdown the expression of GSDMC could inhibit cell proliferation, migration and invasion in PAAD.

### Restoration of GSDMC rescued the inhibition of cell proliferation, migration and invasion induced by GSDMC silencing

In order to test whether the inhibition of cell proliferation and migration is caused by knocking down GSDMC, we re-expressed GSDMC using the lentiviral system in the both PANC-1 and CFPAC-1 cells (Additional file 8: Fig. S8A). According to the CCK8 assay, EdU staining assay, and clone formation assay, we found that the cell proliferation and capability of colony formation were recovered to a certain extent in GSDMC-restored PANC-1 and CFPAC-1 cells after GSDMC silencing, compared with cells with GSDMC silencing (Additional file 8: Fig. S8B–H). Moreover, the cell invasion and migration were also rescued to a certain extent, in GSDMC-restored PANC-1 and CFPAC-1 cells after GSDMC silencing, compared with cells with GSDMC silencing (Additional file 8: Fig. S8G–K). Based on above results, we could conclude that restoration of GSDMC could rescue the inhibition of cell proliferation, migration and invasion after GSDMC silencing.

### Overexpression GSDMC promotes cell proliferation and invasion in PAAD

In order to validate the potential role of GSDMC in PAAD, we performed gain-of-function or loss-of-function analysis. First, we overexpressed GSDMC in PANC-1 and CFPAC-1 cells, the expression of Ki-67 and vimentin was significantly increased and the expression of E-cadherin was significantly reduced, which indicated that overexpression GSDMC can promote PAAD cell proliferation and invasion (Additional file 9: Fig. S9A). Cell viability was measured using the CCK8 assay to determine the effect of GSDMC on PAAD cell proliferation. It was observed that both PANC-1 and CFPAC-1 cells showed a significant increase in their growth curve after transfection with GSDMC (Additional file 9: Fig. S9B, C). Consistent with the abovementioned CCK8 analysis results, overexpression of GSDMC increased more clone numbers than the control group (Additional file 9: Fig. S9D, F). Both in PANC-1 and CFPAC-1 cells showed higher EdU-positive staining when GSDMC overexpressed (Additional file 9: Fig. S9E, H). These results demonstrated that PAAD cell proliferation were facilitated because of the upregulation of GSDMC. Moreover, in the transwell assay, GSDMC overexpression enhanced invasion capacity significantly both in PANC-1 and CFPAC-1 cells (Additional file 9: Fig. S9G, I). In the wound healing assay, the speed of cell migration has been greatly promoted both in PANC-1 and CFPAC-1 cells (Additional file 9: Fig. S9J, K). Taken together, these results suggest that overexpression GSDMC promotes cell proliferation, migration and invasion in PAAD.

### A.443654 inhibits the cell proliferation of PAAD

To investigate the potential therapeutic effects of A.443654 in PAAD, we performed CCK8 assay, clone formation assay, EdU staining assay and cell-cycle analysis in PANC-1 cell line. The CCK8 assay demonstrated that the cell viability of PANC-1 cells showed an increasing trend after treated with A.443654 (Additional file 10: Fig. S10A). The clone formation assay indicated that the number of clones reduced significantly in PANC-1 cells after treated with A.443654 (Additional file 10: Fig. S10B). The EdU incorporation assay suggested that PANC-1 cells showed lower EdU-positive staining after treated with A.443654 (Additional file 10: Fig. S10C). The cell cycle analysis illustrated that the cell proliferation could be suppressed in PAAD after treated with A.443654 (Additional file 10: Fig. S10D, E). Overall, these results indicate that A.443654 could inhibit the cell proliferation of PAAD.

## Discussion

Pyroptosis is a caspase-1-or caspase-11-dependent programmed cell death [17, 18]. It has become increasingly evident that pyroptosis plays an important role in the progression of cancer in recent years. Research has shown that pyroptosis-related genes play a different role in different types of cancer. Cell death releases inflammatory factors to provide tumor cells with a suitable environment for survival [19]. Pyroptosis can promote tumor cell death, making pyroptosis a potential prognostic and therapeutic target in cancer [20]. In HPV infected cervical cancer cells, AIM2 plays a tumor suppressive role by stimulating pyroptosis [10]. However, the role of pyroptosis genes in PAAD is unclear. This study aimed to construct a prognostic model regarding pyroptosis-related genes for diagnosis and prediction of prognosis in patients with PAAD.

In recent years, the search for PAAD biomarkers, prognostic markers, and prognostic models has been gaining increasing attention [21–25]. Patients with PAAD may benefit from these models since they have great ability to predict prognosis. Consistent with previous studies, the prognostic model we constructed still has good performances for predicting the prognosis of patients with PAAD. 

We constructed a prognostic risk model using 5 genes (IL18, CASP4, NLRP1, NLRP2, GSDMC) through univariate Cox and Lasso Cox regression analysis. IL18 is a proinflammatory cytokine that promotes IFN- γ Of secretion [26]. The levels of IL18 are significantly elevated in patients with gouty arthritis [27] and rheumatoid arthritis [28]. CASP4 is a gene involved in encoding a protein involved in immune response and inflammation [29], and studies have shown that decreased expression of CASP4 is associated with poor prognosis in esophageal squamous cell carcinoma [30], but low expression of CASP4 in our prognostic model is more favorable for the survival of PAAD patients. NLRP1 is an innate immune receptor that assembles into an inflammasome to induce pyroptosis in human corneal epithelial cells [31], which is consistent with the manifestations in our constructed model. NLRP2 is highly expressed in renal tubular epithelial cells and plays a role in promoting inflammation [32]. GSDMC is also one of the most important model genes in our study. Pyroptosis is primarily a programmed cell death mediated by Gasdermins (GSDM) [33]. In which GSDM contains molecules such as Gasdermin C (GSDMC), Gasdermin D (GSDMD) [34]. Studies have pointed out that high expression of GSDMC promotes melanoma metastasis [35]. In gastric cancer, GSDMC inhibits tumor cell growth [36]. Overexpression of GSDMC causes poor prognosis in lung adenocarcinoma [37]. An increasing number of studies have shown that the application of GSDMC is extensive. However, studies of GSDMC in PAAD are quite limited. Therefore, we performed cell experiments with GSDMC alone to verify the specific role of GSDMC in PAAD. Our results showed that the growth, proliferation, and migration were inhibited in PAAD cells with silencing of GSDMC, which is consistent with previous studies. Therefore, we speculated that GSDMC contributed to the poor prognosis of pancreatic cancer mainly by promoting tumor growth and migration.

Previous studies showed that PAAD patients who benefit from immunotherapy is limited [38, 39]. Studies have pointed out that the future treatment of pancreatic cancer should be through active combination and adoptive immunotherapy [40]. The combinatorial approach of immunotherapy in conjunction with other modalities is believed to be a promising treatment strategy. Increasing studies have shown that T cells play a key role in immunotherapy [41]. The higher CD8 expression on T cells confers a better prognosis in esophageal, colorectal, and non-small cell lung cancer [42, 43]. The expression of T cells CD8 in low-risk subgroup was higher than that in high-risk subgroup in our study, which is consistent with previous study [44]. In addition, high expression of mast cells resting leads to poor prognosis in hepatocellular carcinoma [45, 46], which is consistent with our study.

In addition, we screened out four potential small molecular compounds, including A.443654, PD.173074, Epothilone.B, and Lapatinib. A.443654 is a well-known Akt serine/threonine kinase inhibitor [47], which is equally potent against Akt1, Akt2, and Akt3 within cells (Ki = 160 pM) [48]. Studies showed that A.443654 could induce apoptosis in chronic lymphocytic leukemia cells in a dose-dependent manner [49]. In addition, A.443654 plays a key role in cells transition to the G2/M phase [50]. Therefore, A.443654 may inhibit PAAD by mediating the cell proliferation, which is consistent with the results of our validation experiments. PD.173074, a small-molecule tyrosine kinase inhibitor, which could interfere with the relevant signaling of fibroblast growth factor [51]. The growth and invasion of Epithelial-mesenchymal transition-induced tumor cells could be inhibited by PD.173074 through EGFR pathway [52]. It has been demonstrated that the FDA approved antitumor drug Epothilone B could improve microtubule stability and promote α-Ability of tubulin polymerization [53]. As an EGFR and HER2 tyrosine kinase inhibitor, Lapatinib is approved by FDA to treat patients with HER2-positive breast cancer [54]. These results indicated that these potential drugs might provide novel insights  into the treatment of patients with PAAD.

However, our study has some limitations. First, GSDMC could promote the proliferation, migration, and invasion of PAAD, but its molecular mechanism is still unknown. Second,  in vivo function of GSDMC in PAAD still need to be explored in the future.

## Conclusions

In this study, we developed a prognostic model based on IL18, CASP4, NLRP1, NLRP2, and GSDMC genes, which effectively predicted the prognosis of PAAD patients. The results suggest that these genes could be potential biomarkers for predicting the overall survival of patients with PAAD. Furthermore, the results of in vitro experiments showed that GSDMC can promote the proliferation, migration and invasion in PAAD cells.

## Supplementary Information

**Additional file 1: Figure S1.** Functional enrichment analyses of gene ontology (GO) and Kyoto Encyclopedia of Genes and Genomes (KEGG). (A) Bubble graph for GO enrichment (the bigger bubble means the more genes enriched, and the increasing depth of red means the differences were more obvious; q-value: the adjusted p-value). (B) KEGG enrichment analysis of differentially expressed genes.

**Additional file 2: Figure S2.** Validation of the pyroptosis-related prognostic model in test set. (A) The patients were divided into two groups according to the threshold of median  risk score. Green represents the low-risk group. Red represents the high-risk group. (B) Kaplan Meier curves showing the overall survival of patients in the high-risk and low-risk groups. (C) Survival status of patients with PAAD in high and low risk groups. Green represents survival. Red represents death. (D) Heatmap showing the expression of the five pyroptosis-related genes from which the model was constructed in the high-and low-risk groups. Pink represents the low-risk group. Bright blue represents the high-risk group. (E) The predictive efficiency of the risk score was verified by the ROC curve.

**Additional file 3: Figure S3.** Risk model independent prognostic analysis. (A) Univariate independent prognosis Cox regression analysis of risk score and indicated clinical characteristics. (B) Multivariate independent prognosis Cox regression analysis of risk score and indicated clinical characteristics.

**Additional file 4: Figure S4.** The correlation of pyroptosis-related prognostic genes and clinical features in the training set. (A, B, C) The correlation of NLRP1 with grade, N stage, T stage. (D) The correlation of NLRP2 with grade. (E) The correlation of GSDMC with stage. (F) The correlation of CASP4 with grade. (G) The correlation of risk score with grade.

**Additional file 5: Figure S5.** GSEA enrichment analysis identifies KEGG pathways associated with high-risk and low-risk groups in the training set (A) and test set (B).

**Additional file 6: Figure S6.** The correlation of immune infiltrating cells with risk scores was determined in the training set. (A) Boxplots represent the level of different types of immune infiltrating cells in high- and low- risk group. (B-F) the correlation between the risk score and immune infiltrating cells was further examined by Spearman correlation analysis. *p < 0.05, ***p < 0.001.

**Additional file 7: Figure S7.** Kaplan-Meier curves of the prognostic pyroptosis-related genes in the training set. The survival curves of NLRP1 (A), NLRP2 (B), GSDMC (C), IL18 (D), CASP4 (E).

**Additional file 8: Figure S8.** Restoration of GSDMC rescued the inhibition of cell proliferation, migration and invasion induced by GSDMC silencing. (A) PANC-1 (left panel) and CFPAC-1 (right panel) cells were subjected to immunoblotting as indicated. (B, C) Cell growth of PANC-1 cells (B) and CFPAC-1 cells (C) were measured by CCK-8 assay. *P < 0.05, **P < 0.01 by two-way ANOVA. (D, F) Clone formation of PANC-1 cells (D) and CFPAC-1 cells (F) were measured as indicated. *P < 0.05, **P < 0.01 by one-way ANOVA. (E, H) Representative image of Edu staining and quantitative analysis of Edu staining in PANC-1 cells (E) and CFPAC-1 cells (H). *P < 0.05, **P < 0.01, ***P < 0.001 by one-way ANOVA. (G, I) Cell invasion of indicated PANC-1 cells (G) and CFPAC-1 cells (I) was measured by trans-well assay. **P < 0.01, ***P < 0.001 by one-way ANOVA. (J, K) Cell migration of  PANC-1 cells (J) and CFPAC-1 cells (K) was measured by wound-healing assay as indicated. *P < 0.05, **P < 0.01, ***P < 0.001 by one-way ANOVA.

**Additional file 9: Figure S9.** Overexpression GSDMC promotes cell proliferation and invasion in PAAD. (A)PANC-1 (left panel) and CFPAC-1 (right panel) cells were subjected to immunoblotting as indicated. (B, C) Cell growth of PANC-1 cells (B) and CFPAC-1 cells (C) were measured by CCK-8 assay.  *P < 0.05, **P < 0.01 by two-way ANOVA. (D, F) Clone formation of PANC-1 cells (D) and CFPAC-1 cells (F) were measured as indicated ***P < 0.001 by a two-tailed unpaired t-test. (E, H)  Representative image of Edu staining and quantitative analysis of Edu staining in PANC-1 cells (E) and CFPAC-1 cells (H). **P < 0.01 by a two-tailed unpaired t-test. (G, I) Cell invasion of indicated PANC-1 cells (G) and CFPAC-1 cells (I) was measured by trans-well assay.  *P < 0.05, ***P < 0.001 by a two-tailed unpaired t-test. (J, K) Cell migration of  PANC-1 cells (J) and CFPAC-1 cells (K) was measured by wound-healing assay as indicated. *P < 0.05 by a two-tailed unpaired t-test.

**Additional file 10: **Figure S10. A.443654 inhibits the cell proliferation of PAAD. (A) Cell viability of PANC-1 cell line treated with DMSO and 50 nM A.443654, respectively. *P < 0.05, **P < 0.01 by a two-tailed unpaired t-test. (B) Colony formation assay of PANC-1 cells treated with DMSO and 50 nM A.443654, respectively. **P < 0.01 by a two-tailed unpaired t-test. (C) Edu assay of PANC-1 cells treated with DMSO and 50 nM A.443654, respectively. *P < 0.05 by a two-tailed unpaired t-test. (D) Representative cell-cycle analysis of PANC-1 cells treated with DMSO and 50 nM A.443654, respectively. (E) Quantification of cell-cycle results in PANC-1 cells. **P < 0.01, ***P < 0.001 by a two-tailed unpaired t-test.

**Additional file 11:** Drugs with significant differences in IC50 values between high-risk and low-risk groups.

## Data Availability

All data and R script in this study are available from the corresponding author upon reasonable request. All authors read and approved the final manuscript. Publicly available datasets were analyzed in this study, these can be found in The Cancer Genome Atlas (https://portal.gdc.cancer.gov/), Genotype-Tissue Expression (https://www.gtexportal.org/home/), and Gene Expression Omnibus (GSE71729, GSE57495).
