# Correction: System analysis based on the pyroptosis-related genes identifies GSDMC as a novel therapy target for pancreatic adenocarcinoma

**DOI:** 10.1186/s12967-025-07200-z

**Published:** 2025-10-20

**Authors:** Cheng Yan, Yandie Niu, Feng Li, Wei Zhao, Liukai Ma

**Affiliations:** School of Pharmacy, Key Laboratory of Nano-Carbon Modified Film Technology of Henan Province, Diagnostic Laboratory of Animal Diseases, Xinxiang University, Xinxiang, Henan 453000 China

**Correction: J Transl Med 20, 455 (2022)**

10.1186/s12967-022-03632-z

Following publication of the original article [1], the authors reported inadvertent image misuses in Figure 8 and errors in the Figure Legend. Specifically, Figures 8E (EdU Assay – CFPAC-1), 8H (EdU Assay – PANC-1) and 8I (Transwell Assay – PANC-1) were misused.

The incorrect version of Figure 8 and Legend was:

**Fig. 8:**
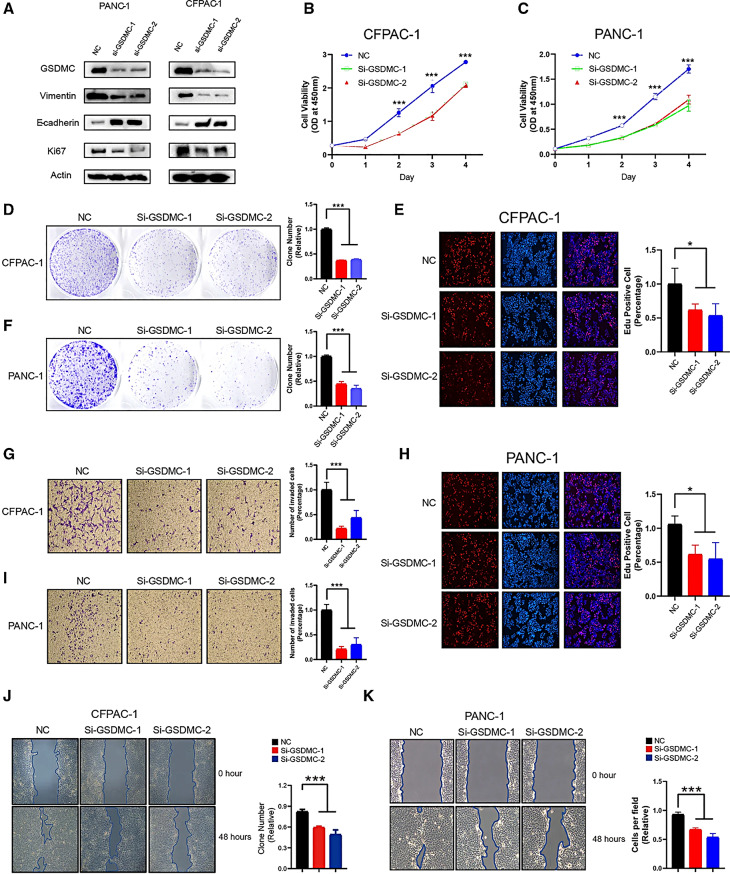
Knockdown of GSDMC inhibited PAAD cell proliferation and migration. **A** Western blot results showing the expression levels of indicated proteins in PANC-1 (left panel) or CFPAC-1 (right panel) cell line transfected with scrambled or two independent siRNA targeting GSDMC, respectively. **B**, **C** Cell viability was determined by CCK8 assay in the PANC-1 (B) and CFPAC-1 (C) cell lines transfected with either scrambled or two independent GSDMC siRNA targeting GSDMC, respectively. ***p < 0.001 by one-way ANOVA. **D**, **F** Colony formation assay of CFPAC-1 (D) and PANC-1 (F) cell lines with either scrambled or two independent siRNA targeting GSDMC, respectively. ***p < 0.001 by one-way ANOVA. **E**, **H** Edu assay to show the cell proliferation of PANC-1 (E) and CFPAC-1 (H) cell lines transfected with either scrambled or two independent siRNA targeting GSDMC, respectively. *p < 0.05 by one-way ANOVA. **J**, **K** Wound healing assay of PANC-1 (J) and CFPAC-1 (K) cell migration capability following transfected with scrambled or two independent siRNA targeting GSDMC, respectively. ***p < 0.001 by one-way ANOVA

The correct Figure 8 and Legend are:

**Fig. 8:**
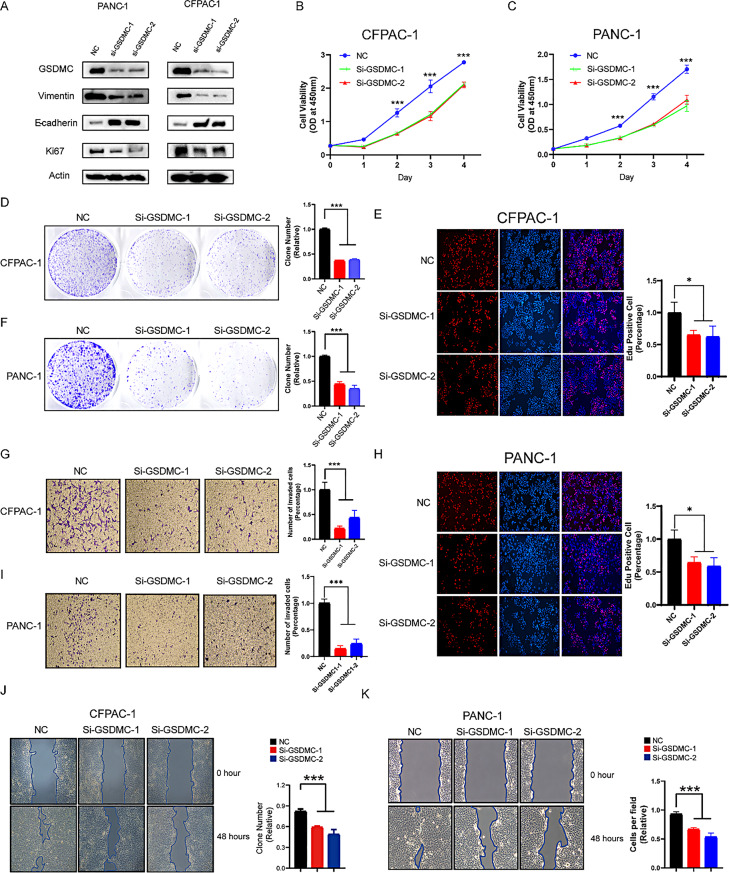
Knockdown of GSDMC inhibits PAAD cell proliferation and migration. **A** Western blot analysis showing GSDMC expression in PANC-1 (left) and CFPAC-1 (right) cells transfected with scrambled control or two independent siRNAs targeting GSDMC. **B**,** C** Cell viability of CFPAC-1 (**B**) and PANC-1 (**C**) cells was measured by CCK-8 assay. ***p < 0.001 by one-way ANOVA. **D**,** F** Colony formation assays of CFPAC-1 (**D**) and PANC-1 (**F**) cells with or without GSDMC knockdown. ***p < 0.001 by one-way ANOVA. **E**,** H** EdU assays showing reduced proliferation of CFPAC-1 (**E**) and PANC-1 (**H**) cells upon GSDMC knockdown. *p < 0.05 by one-way ANOVA. **G**,** I** Transwell assays showing decreased migration in CFPAC-1 (**G**) and PANC-1 (**I**) cells following siRNA-mediated knockdown of GSDMC. *p < 0.05 by one-way ANOVA. **J**,** K** Wound healing assays demonstrating reduced migration of CFPAC-1 (**J**) and PANC-1 (**K**) cells after GSDMC depletion. ***p < 0.001 by one-way ANOVA

The original article [1] has been updated.
