# Supplementary figures and images for: System analysis based on the pyroptosis-related genes identifies GSDMC as a novel therapy target for pancreatic adenocarcinoma
Source: J Transl Med. 2022 Oct 5;20:455. doi: 10.1186/s12967-022-03632-z (PMC9533512; doi:10.1186/s12967-022-03632-z)

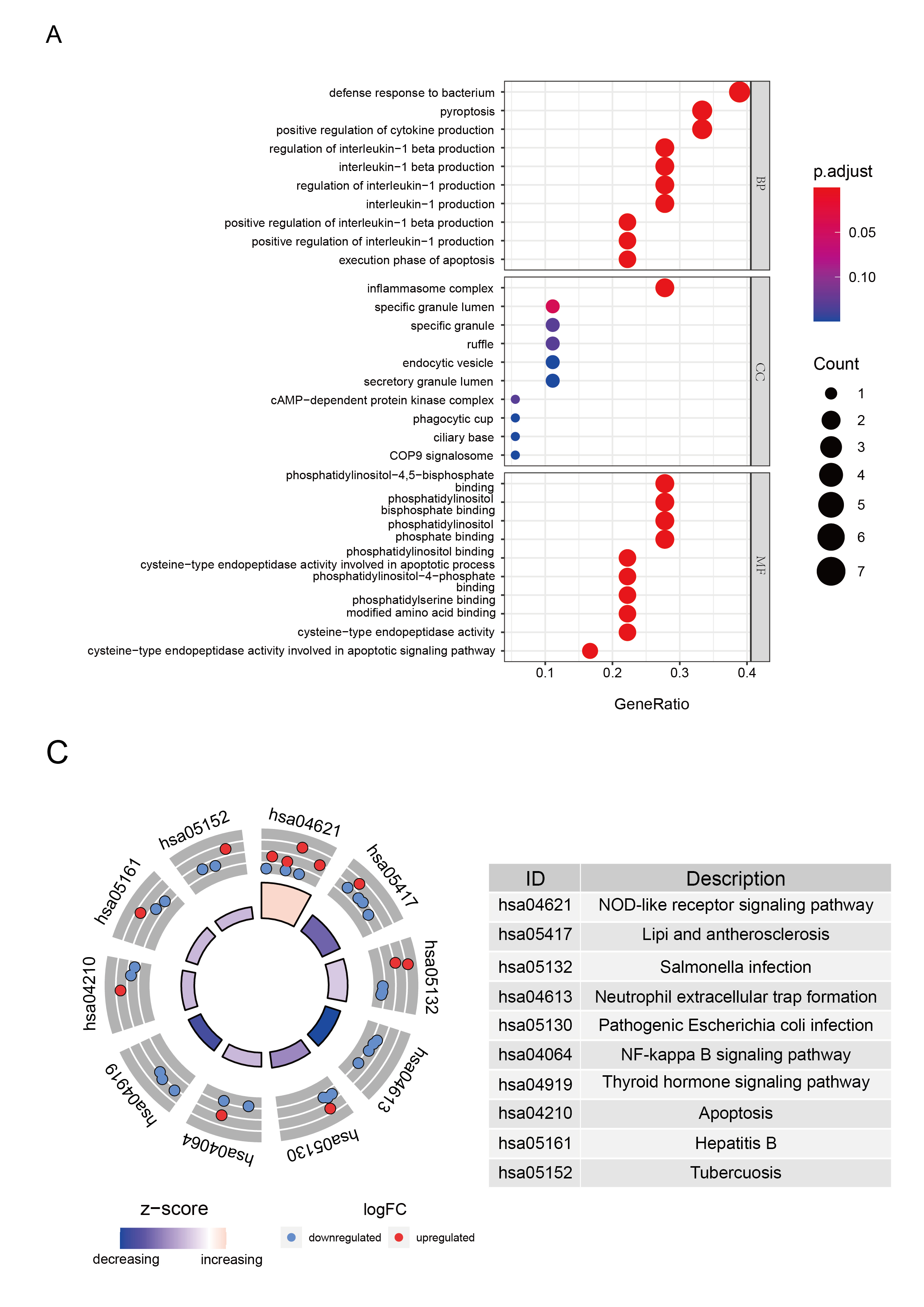

Supplement: Supplementary file 1 — Additional file 1: Figure S1. Functional enrichment analyses of gene ontology (GO) and Kyoto Encyclopedia of Genes and Genomes (KEGG). (A) Bubble graph for GO enrichment (the bigger bubble means the more genes enriched, and the increasing depth of red means the differences were more obvious; q-value: the adjusted p-value). (B) KEGG enrichment analysis of differentially expressed genes. [file 12967_2022_3632_MOESM1_ESM.jpg]

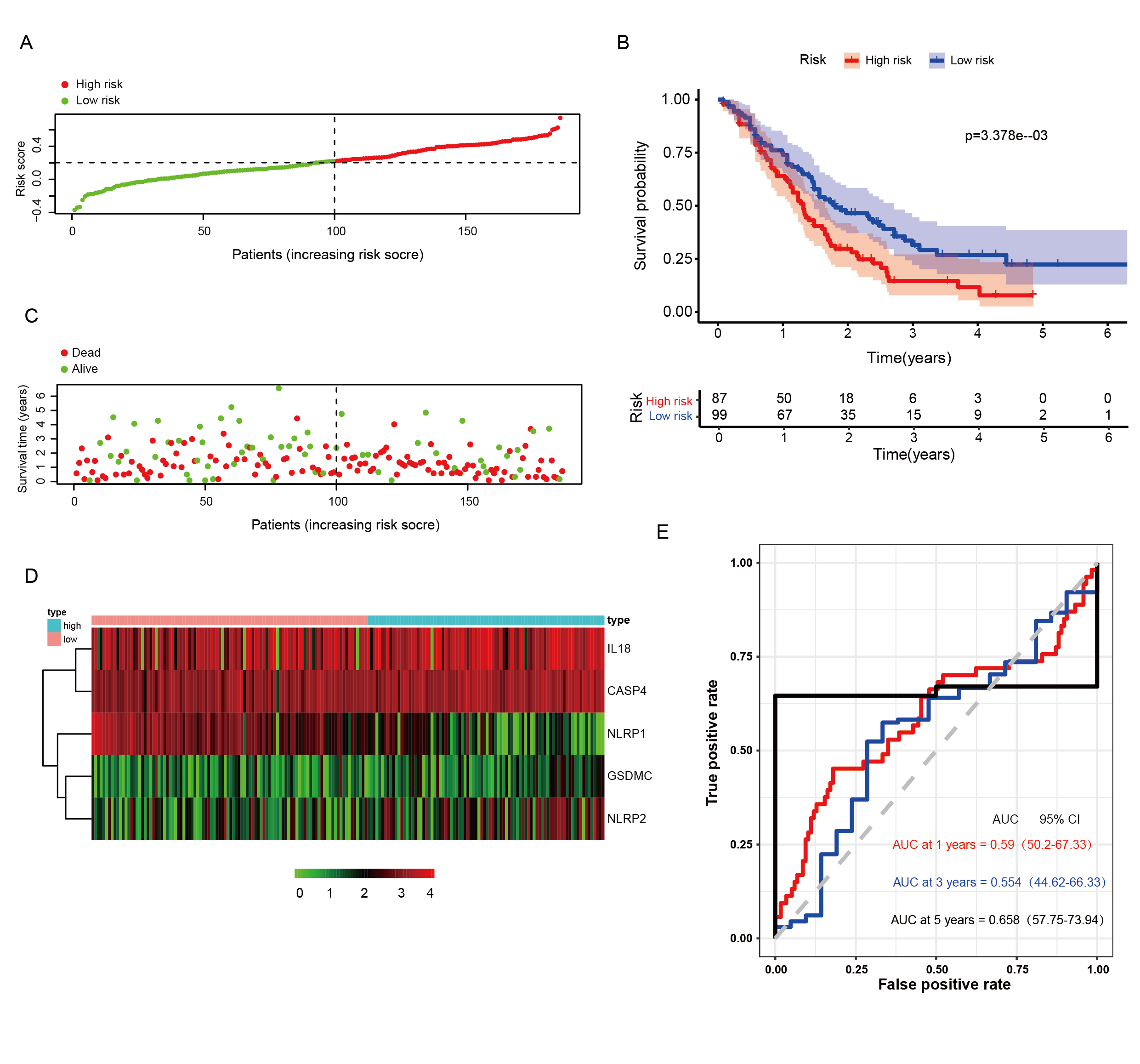

Supplement: Supplementary file 2 — Additional file 2: Figure S2. Validation of the pyroptosis-related prognostic model in test set. (A) The patients were divided into two groups according to the threshold of median risk score. Green represents the low-risk group. Red represents the high-risk group. (B) Kaplan Meier curves showing the overall survival of patients in the high-risk and low-risk groups. (C) Survival status of patients with PAAD in high and low risk groups. Green represents survival. Red represents death. (D) Heatmap showing the expression of the five pyroptosis-related genes from which the model was constructed in the high-and low-risk groups. Pink represents the low-risk group. Bright blue represents the high-risk group. (E) The predictive efficiency of the risk score was verified by the ROC curve. [file 12967_2022_3632_MOESM2_ESM.jpg]

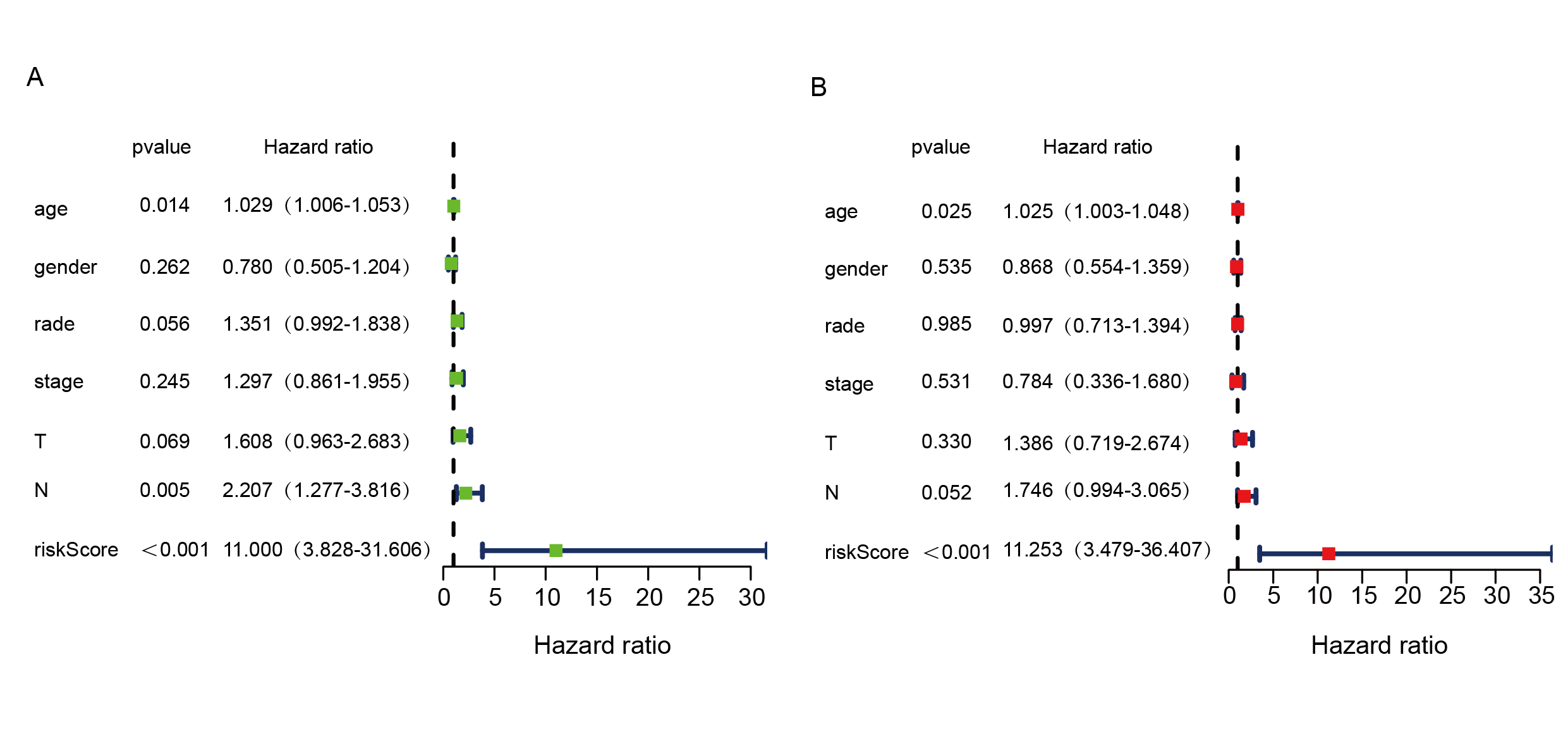

Supplement: Supplementary file 3 — Additional file 3: Figure S3. Risk model independent prognostic analysis. (A) Univariate independent prognosis Cox regression analysis of risk score and indicated clinical characteristics. (B) Multivariate independent prognosis Cox regression analysis of risk score and indicated clinical characteristics. [file 12967_2022_3632_MOESM3_ESM.jpg]

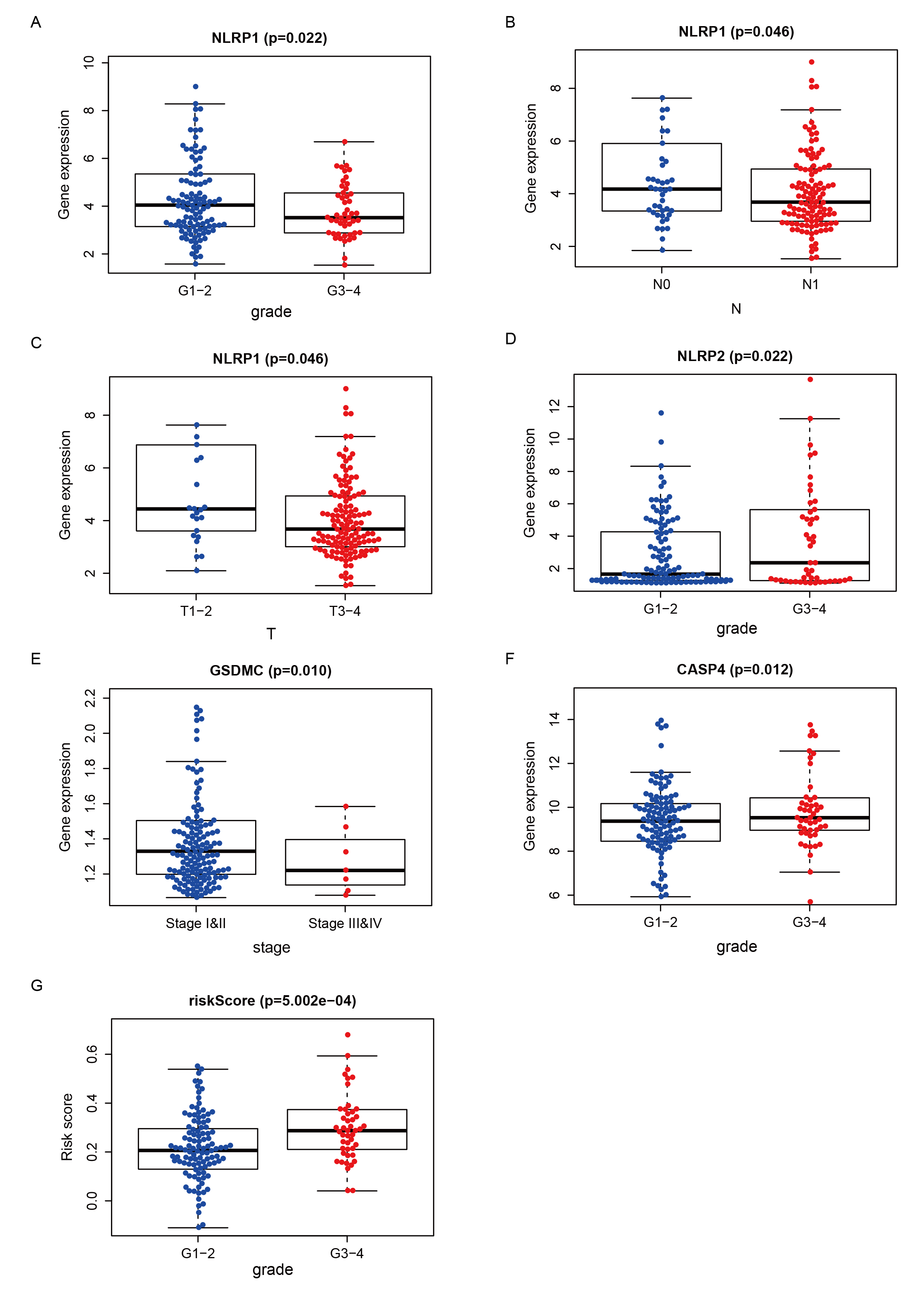

Supplement: Supplementary file 4 — Additional file 4: Figure S4. The correlation of pyroptosis-related prognostic genes and clinical features in the training set. (A, B, C) The correlation of NLRP1 with grade, N stage, T stage. (D) The correlation of NLRP2 with grade. (E) The correlation of GSDMC with stage. (F) The correlation of CASP4 with grade. (G) The correlation of risk score with grade. [file 12967_2022_3632_MOESM4_ESM.jpg]

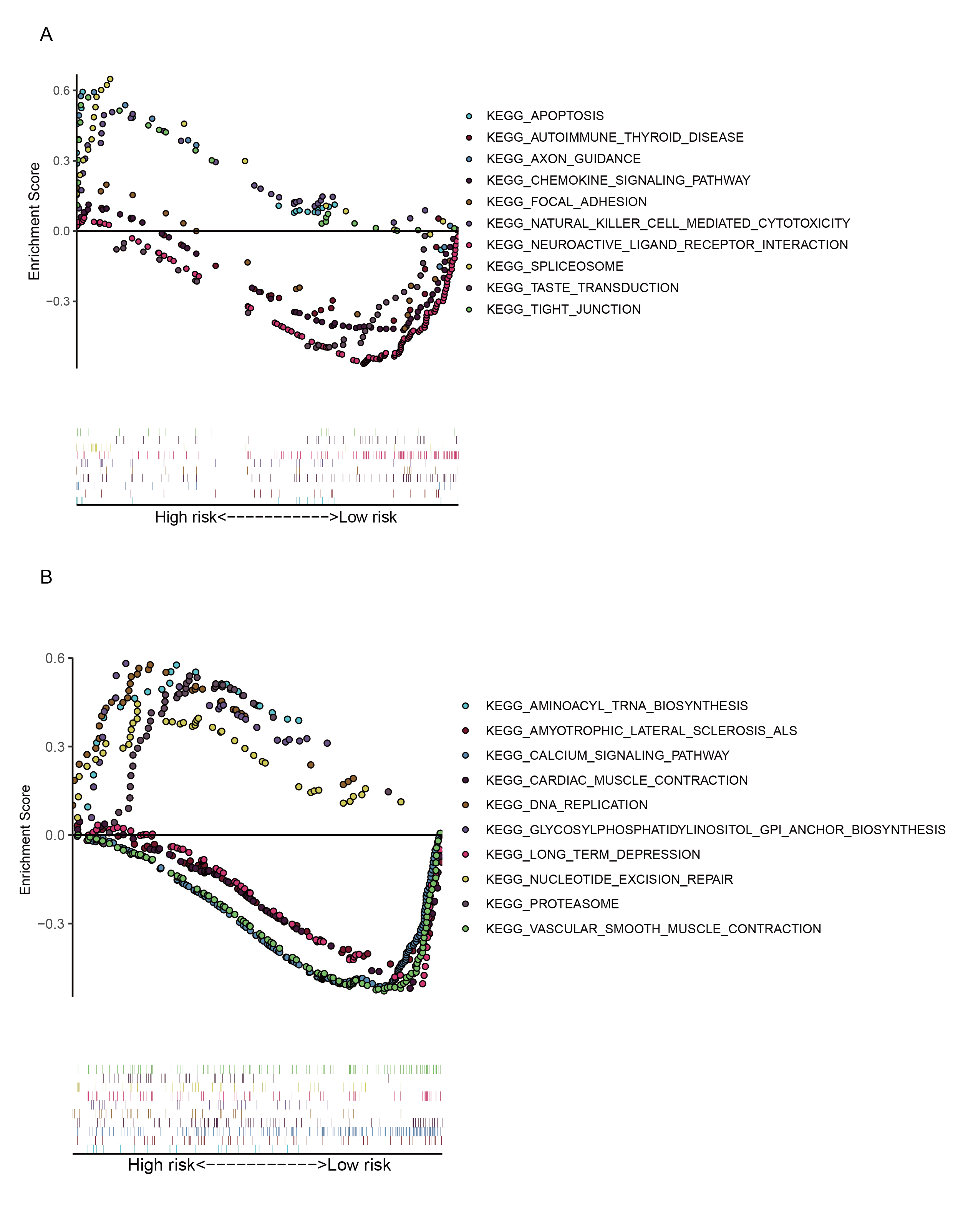

Supplement: Supplementary file 5 — Additional file 5: Figure S5. GSEA enrichment analysis identifies KEGG pathways associated with high-risk and low-risk groups in the training set (A) and test set (B). [file 12967_2022_3632_MOESM5_ESM.jpg]

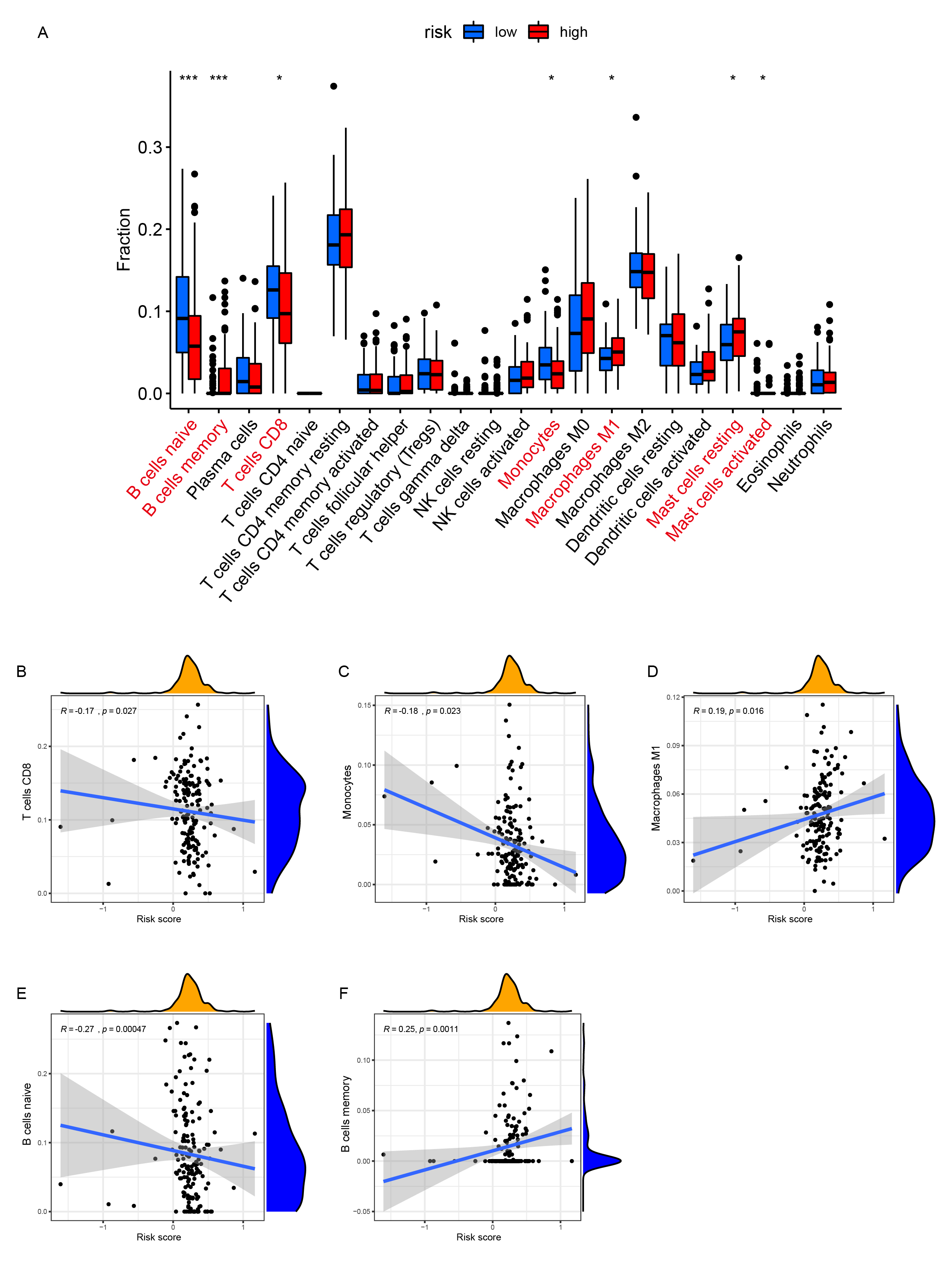

Supplement: Supplementary file 6 — Additional file 6: Figure S6. The correlation of immune infiltrating cells with risk scores was determined in the training set. (A) Boxplots represent the level of different types of immune infiltrating cells in high- and low- risk group. (B-F) the correlation between the risk score and immune infiltrating cells was further examined by Spearman correlation analysis. *p < 0.05, ***p < 0.001. [file 12967_2022_3632_MOESM6_ESM.jpg]

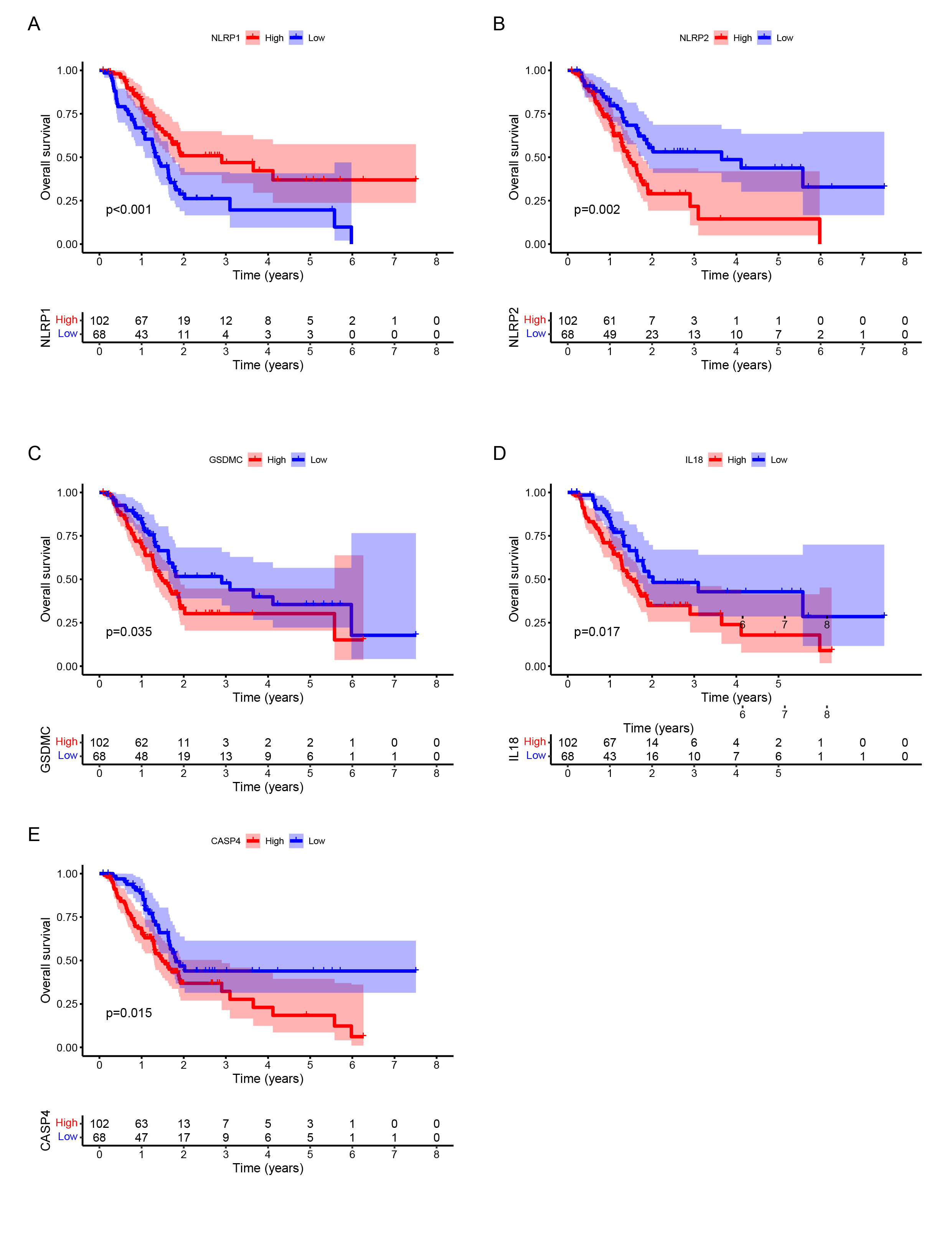

Supplement: Supplementary file 7 — Additional file 7: Figure S7. Kaplan-Meier curves of the prognostic pyroptosis-related genes in the training set. The survival curves of NLRP1 (A), NLRP2 (B), GSDMC (C), IL18 (D), CASP4 (E). [file 12967_2022_3632_MOESM7_ESM.jpg]

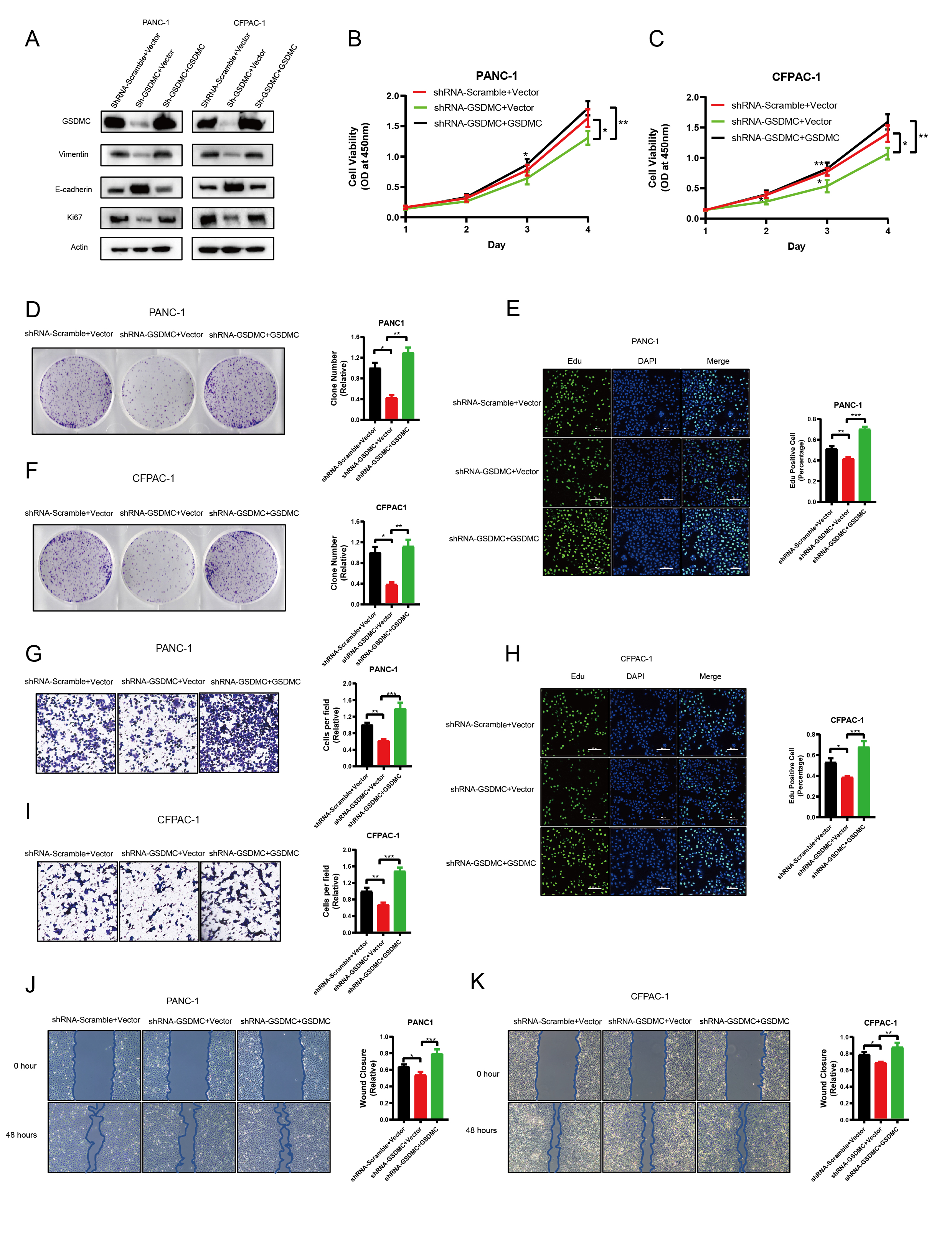

Supplement: Supplementary file 8 — Additional file 8: Figure S8. Restoration of GSDMC rescued the inhibition of cell proliferation, migration and invasion induced by GSDMC silencing. (A) PANC-1 (left panel) and CFPAC-1 (right panel) cells were subjected to immunoblotting as indicated. (B, C) Cell growth of PANC-1 cells (B) and CFPAC-1 cells (C) were measured by CCK-8 assay. *P < 0.05, **P < 0.01 by two-way ANOVA. (D, F) Clone formation of PANC-1 cells (D) and CFPAC-1 cells (F) were measured as indicated. *P < 0.05, **P < 0.01 by one-way ANOVA. (E, H) Representative image of Edu staining and quantitative analysis of Edu staining in PANC-1 cells (E) and CFPAC-1 cells (H). *P < 0.05, **P < 0.01, ***P < 0.001 by one-way ANOVA. (G, I) Cell invasion of indicated PANC-1 cells (G) and CFPAC-1 cells (I) was measured by trans-well assay. **P < 0.01, ***P < 0.001 by one-way ANOVA. (J, K) Cell migration of PANC-1 cells (J) and CFPAC-1 cells (K) was measured by wound-healing assay as indicated. *P < 0.05, **P < 0.01, ***P < 0.001 by one-way ANOVA. [file 12967_2022_3632_MOESM8_ESM.jpg]

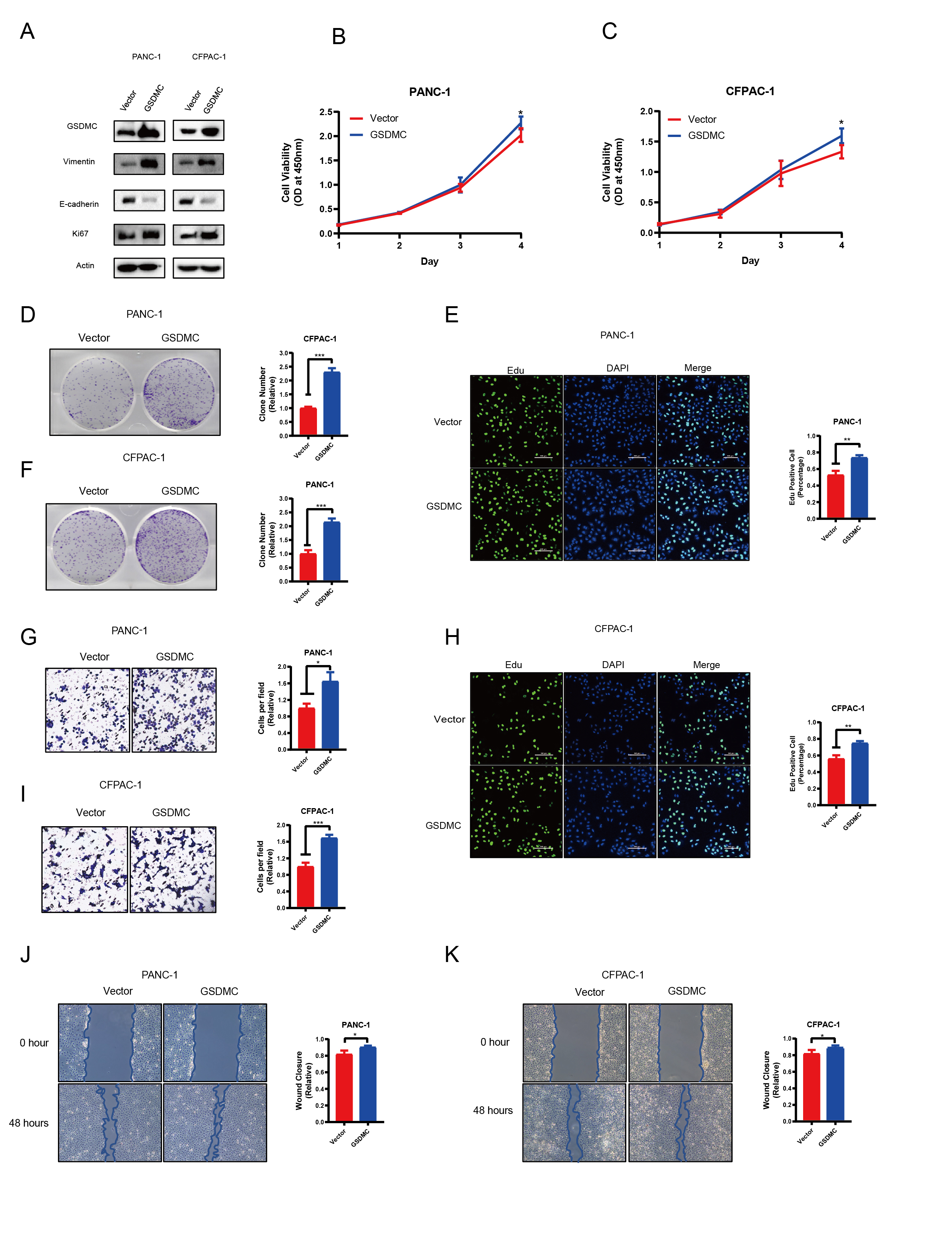

Supplement: Supplementary file 9 — Additional file 9: Figure S9. Overexpression GSDMC promotes cell proliferation and invasion in PAAD. (A)PANC-1 (left panel) and CFPAC-1 (right panel) cells were subjected to immunoblotting as indicated. (B, C) Cell growth of PANC-1 cells (B) and CFPAC-1 cells (C) were measured by CCK-8 assay. *P < 0.05, **P < 0.01 by two-way ANOVA. (D, F) Clone formation of PANC-1 cells (D) and CFPAC-1 cells (F) were measured as indicated ***P < 0.001 by a two-tailed unpaired t-test. (E, H) Representative image of Edu staining and quantitative analysis of Edu staining in PANC-1 cells (E) and CFPAC-1 cells (H). **P < 0.01 by a two-tailed unpaired t-test. (G, I) Cell invasion of indicated PANC-1 cells (G) and CFPAC-1 cells (I) was measured by trans-well assay. *P < 0.05, ***P < 0.001 by a two-tailed unpaired t-test. (J, K) Cell migration of PANC-1 cells (J) and CFPAC-1 cells (K) was measured by wound-healing assay as indicated. *P < 0.05 by a two-tailed unpaired t-test. [file 12967_2022_3632_MOESM9_ESM.jpg]

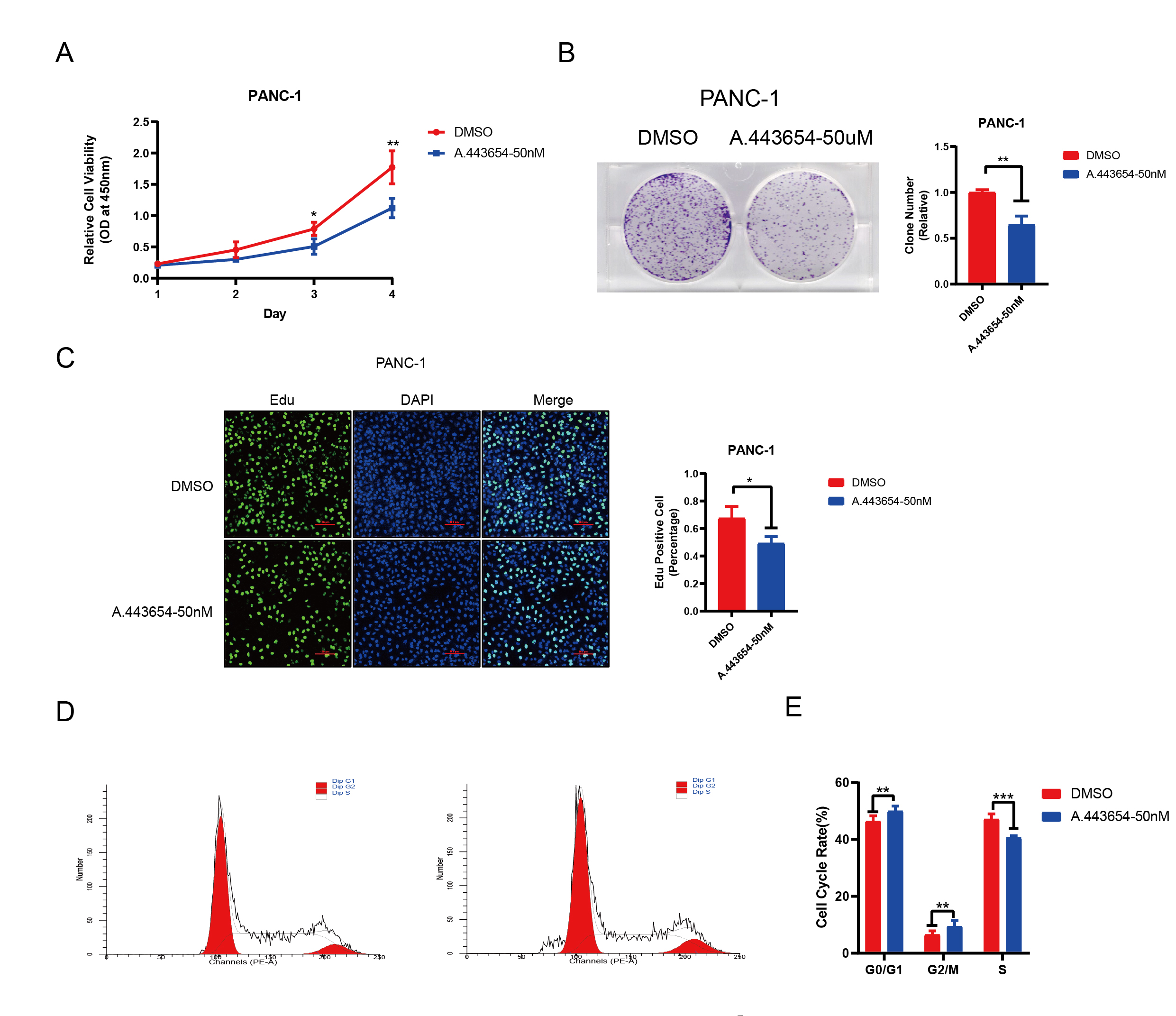

Supplement: Supplementary file 10 — Additional file 10: Figure S10. A.443654 inhibits the cell proliferation of PAAD. (A) Cell viability of PANC-1 cell line treated with DMSO and 50 nM A.443654, respectively. *P < 0.05, **P < 0.01 by a two-tailed unpaired t-test. (B) Colony formation assay of PANC-1 cells treated with DMSO and 50 nM A.443654, respectively. **P < 0.01 by a two-tailed unpaired t-test. (C) Edu assay of PANC-1 cells treated with DMSO and 50 nM A.443654, respectively. *P < 0.05 by a two-tailed unpaired t-test. (D) Representative cell-cycle analysis of PANC-1 cells treated with DMSO and 50 nM A.443654, respectively. (E) Quantification of cell-cycle results in PANC-1 cells. **P < 0.01, ***P < 0.001 by a two-tailed unpaired t-test. [file 12967_2022_3632_MOESM10_ESM.jpg]
